# Unveiling the diversity, ecology, and biotechnological potential of culturable marine yeasts in Western Mediterranean coastal ecosystems

**DOI:** 10.3897/imafungus.17.182209

**Published:** 2026-05-29

**Authors:** Alejandra Martínez-Rodrigo, Andrés Núñez, Alejandro Franco, Marisa Madrid, Jero Vicente-Soler, José Cansado, Teresa Soto

**Affiliations:** 1 Yeast Physiology Group, Department of Genetics and Microbiology, Campus de Excelencia Internacional de Ámbito Regional (CEIR) Campus Mare Nostrum, Universidad de Murcia, 30100 Murcia, Spain Universidad de Murcia Murcia Spain

**Keywords:** Biotechnological potential, coastal microbiology, culturable marine yeasts, ecological function, spatiotemporal distribution, yeast diversity

## Abstract

Coastal ecosystems are dynamic environments characterized by strong seasonal variability in physicochemical parameters and biological communities. In the western Mediterranean, the Region of Murcia (southeastern Spain) is characterized by notable biodiversity and ecological heterogeneity, encompassing diverse habitats, including rocky shores, sandy beaches, seagrass meadows, and the Mar Menor, one of Europe’s largest hypersaline lagoons. Within these systems, marine yeasts play critical ecological roles in organic matter recycling and produce metabolites of biotechnological and medical interest. This study presents the first comprehensive survey of culturable marine yeasts from the Mediterranean coast of the Region of Murcia. From seawater and sediment samples, 415 strains were identified via internal transcribed spacer (ITS) and large subunit (LSU) rDNA sequencing, revealing 90 species across 44 genera, including 78 known taxa and 12 potentially novel ones. Most isolates were assigned to the phylum *Ascomycota* (62%), primarily distributed among the classes *Pichiomycetes*, *Dothideomycetes*, *Saccharomycetes*, and *Dipodascomycetes*, whereas *Basidiomycota* accounted for the remaining 38%, mainly comprising the classes *Tremellomycetes* and *Microbotryomycetes*. The genera most represented in both seawater and sediment were *Rhodotorula* and *Candida*, and approximately 10% of the isolates corresponded to stress-tolerant black yeast-like fungi (*Aureobasidium* spp., *Hortaea werneckii*, *Exophiala oligosperma*, and *Zalaria alba*), a group notable for its taxonomic novelty and valuable biotechnological traits. Culturable yeast assemblages displayed pronounced spatiotemporal variability, with diversity generally increasing during milder seasons and in areas influenced by anthropogenic activity, suggesting the combined effect of natural gradients and human-driven alterations. Representative strains exhibited broad enzymatic capabilities, producing extracellular cellulases, proteases, xylanases, amylases, chitinases, and pectinases, underscoring their ecological role in organic matter turnover and nutrient cycling. The findings provide novel insights into the taxonomic composition and metabolic potential of marine yeasts recovered from the Mediterranean Sea and set the basis for their future exploitation in industrial and environmental biotechnology.

## Introduction

Marine environments harbor a largely untapped diversity of microorganisms with potential biotechnological and commercial applications. While bacteria are the most studied marine microbes, yeasts represent a minor (<1%) yet ecologically significant component of marine microbiomes (Sen et al. 2022). Yeasts are a polyphyletic group of unicellular or dimorphic fungi that reproduce predominantly by budding or fission (Allard and Moseley 2019; Mitchison-Field et al. 2019). Rather than constituting a single phylogenetic lineage, they represent a fungal lifestyle that has independently emerged across distinct lineages in *Dikarya*, with more than 1,300 species described by the end of the twentieth century and a broad environmental distribution (Boekhout et al. 2022). Marine yeasts, isolated from seawater, sediments, and marine organisms, are distinguished by their ability to grow in seawater-based media. Although the terms “obligate” and “facultative” marine yeasts have been widely employed in the literature, the existence of strictly obligate marine yeasts, in a physiological sense, remains unsubstantiated. Most strains recovered from marine environments are halotolerant rather than truly halophilic and do not exhibit an absolute requirement for seawater or elevated salinity for growth. Accordingly, the distinction between terrestrial and marine yeasts is increasingly interpreted as an ecological and evolutionary continuum shaped by environmental selection pressures, rather than as a discrete binary classification (Kutty and Philip 2008; Zaky et al. 2014; Gonçalves et al. 2022). Research over the past decades, including high-throughput sequencing, has revealed more than 200 species in marine ecosystems (Jones et al. 2015, 2019; Fotedar et al. 2022). Many, including melanized yeast-like fungi historically referred to as “black yeast”, have adapted to extreme marine conditions, tolerating osmotic stress, hydrostatic pressure, temperature fluctuations, and nutrient limitation, which enables survival across diverse habitats from coastal nutrient-rich zones to deep-sea sediments (Fotedar et al. 2022). These organisms play critical ecological roles in organic matter recycling and produce metabolites of biotechnological and medical interest, including enzymes, polysaccharides, lipids, antimicrobials, and anticancer compounds, as well as participating in bioremediation (Zaky et al. 2014; Chi et al. 2016). Coastal environments enriched by anthropogenic inputs typically support higher yeast abundance and diversity, whereas abundance decreases in high-salinity or nutrient-poor habitats (Bass et al. 2007; Fotedar et al. 2022). Their resilience and niche specialization have fostered the evolution of novel bioactive compounds, highlighting marine yeasts as valuable reservoirs for industrial and biotechnological applications (Kutty and Philip 2008; Asseri et al. 2025).

The Mediterranean coast of Spain, particularly the Region of Murcia (“Costa Cálida”), extends for ~250 km and encompasses diverse marine environments, including rocky shores, sandy beaches, recreational areas, commercial and fishing ports, seagrass meadows, and the Mar Menor (~135 km^2^), one of the largest hypersaline lagoons in Europe, protected under the Ramsar Convention on Wetlands of International Importance, Natura 2000, and Special Protection Area (SPA) frameworks (Pérez-Ruzafa et al. 2020). These ecosystems provide essential ecological services, support regional fisheries, and are under increasing pressure from climate change and human activity (Velasco et al. 2006; Pérez-Ruzafa et al. 2011, 2020). Despite the biological richness of these environments, no studies since the preliminary surveys conducted in 1984 have comprehensively investigated yeast diversity or explored their biotechnological potential (Marín et al. 1984). The semi-arid Mediterranean climate, characterized by high annual solar radiation and low precipitation, shapes a fragile coastal ecosystem that is currently under significant anthropogenic pressure from tourism, urban development, and intensive agriculture (Velasco et al. 2006; Conesa and Jiménez-Cárceles 2007; Roson and Sartori 2014; Pérez-Ruzafa et al. 2020). This study provides the first comprehensive assessment of the diversity and spatiotemporal distribution of culturable yeasts along the Murcian Mediterranean coast, revealing high taxonomic richness with spatial and seasonal patterns shaped by physicochemical conditions and anthropogenic influences. Many isolates exhibited notable stress tolerance and broad enzymatic activity, underscoring both the ecological relevance and biotechnological potential of this underexplored microbial reservoir.

## Materials and methods

### Study area, sampling, and environmental parameters

**Figure 1.**
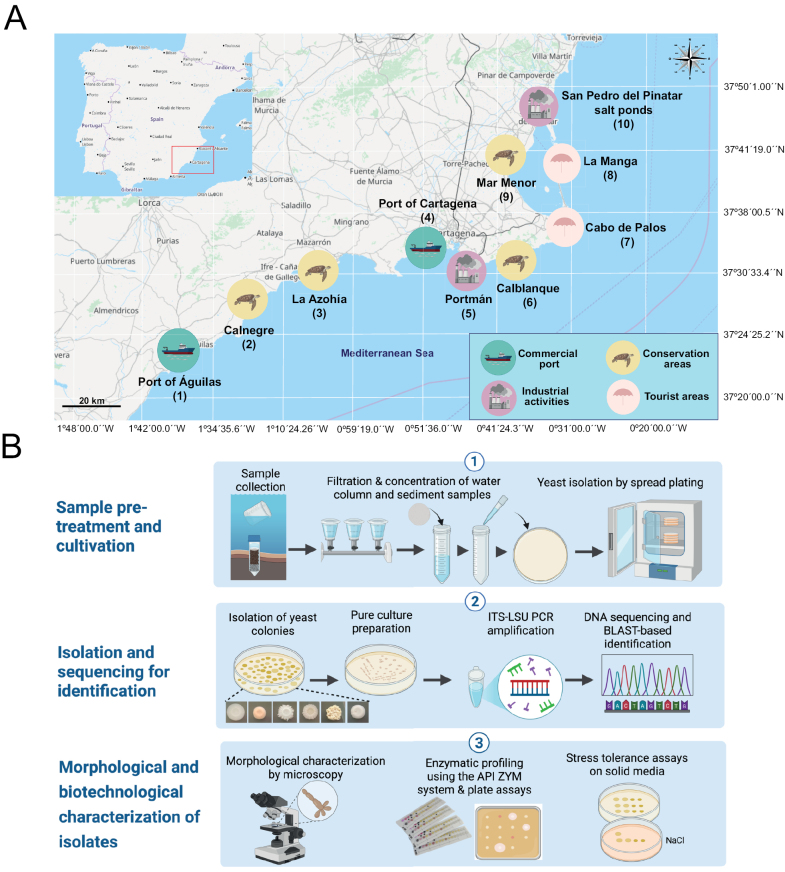
Sampling map and overview of the methodological workflow. **A**. Geographic location of the sampling sites along the Mediterranean coastline in the Region of Murcia, southeastern Spain. The sites (1–10) are classified by their predominant environmental use: commercial port (sites 1 and 4), conservation area (sites 2, 3, 6, and 9), industrial activities (sites 5 and 10), and tourist area (sites 7 and 8). Map data obtained from © OpenStreetMap contributors. **B**. Workflow for marine yeast isolation and characterization, including culturing, isolation, taxonomic identification, and physiological profiling (created with BioRender).

Field sampling was carried out along ~250 km of the Murcia coastline (southeastern Spain, western Mediterranean Sea) across four consecutive seasons, from autumn 2024 to summer 2025. Seasonal seawater and sediment samples were collected at ten sites (1–10) representing contrasting geomorphological features and gradients of environmental and anthropogenic influence. These sampling points included commercial and fishing ports (site 1: Port of Águilas; site 4: Port of Cartagena); marine reserves or regional parks of ecological importance (site 2: Calnegre; site 3: La Azohía; site 6: Regional Park of Calblanque-Monte de las Cenizas-Peña del Águila, hereinafter referred to as “Calblanque”); areas with mining influence (site 5: Portmán); recreational rocky beaches (site 7: Cabo de Palos); sandy beaches (site 8: La Manga); coastal salt lagoons with some anthropogenic influence (site 9: Mar Menor); and salt ponds characterized by extreme hypersalinity (site 10: Salt Ponds of San Pedro del Pinatar) (Fig. 1A).

At each sampling site, the same number of seawater and sediment samples were collected in parallel. Specifically, water (3 L) and sediment (100 g) were collected in triplicate at 1 m offshore and at the surface level using sterile glass containers. These samples were stored in the dark at 4 °C until they were processed in the laboratory. *In situ* measurements of seawater temperature, pH, dissolved oxygen (DO), specific conductance (SPC), total dissolved solids (TDS), and salinity were conducted using a portable multiparameter analyzer (XS REVIO, G-PCREVIO-3, Italy). Subsamples were used for the determination of chlorophyll-a (Chl-a) and inorganic nutrients (NO_3_^–^, NO_2_^–^, PO_4_^3–^, and NH_4_^+^) analyzed using spectrophotometric methods (Hansen 2007). For Chl-a, 1 L of seawater was filtered (GF/C filters, Whatman), 5 mL of acetone (90%) was added to the filters, and the concentration was spectrophotometrically assessed according to Jeffrey and Humphrey (1975). To estimate the granulometry of sediments, the wet samples were dried at 80 °C for 24 h. The resulting dry sediment was then sieved into size fractions using a series of stainless-steel mesh sieves with nominal openings of 2–1 mm, 1–0.5 mm, 0.5–0.25 mm, 0.25–0.063 mm, and < 0.063 mm. The finest fraction (<0.063 mm), corresponding to silt and clay, was retained for subsequent analyses of nitrogen (N), phosphorus (P), sulfur (S), carbon (C), and hydrogen (H) using an elemental analyzer (CHNS method) at the Scientific and Technical Research Area (ACTI), University of Murcia. This sieving procedure follows the method described by Liu and Lee (2006), which enables the separation of sediment particles into standardized size classes suitable for granulometric and geochemical studies.

### Yeast isolation

The yeast isolation procedure from water and sediment samples is summarized in Fig. 1B. Yeast isolation was primarily based on a membrane filtration approach adapted from Zaky et al. (2014) and Mitchison-Field et al. (2019). Briefly, 1 L of water was filtered through sterile nitrocellulose membranes (0.45 μm pore size; 47 mm diameter, Millipore) using a sterilized filtration unit. For sediment samples, 10 g were suspended in 200 mL of sterile saline solution (0.85%) and, after stirring at 150 rpm for 30 min at 25 °C, were filtered following the same procedure as for water. The process was replicated three times for each sampling site. Filtered samples were concentrated 100- or 500-fold through resuspension of the retained particles in 2 mL of sterile saline solution. The resulting concentrate was immediately plated in duplicate (200 µL/plate) onto five different types of culture media, each providing distinct carbon sources to support the growth of diverse microbial communities: YPD (1% yeast extract, 2% peptone, 2% dextrose, 2% agar), PDA (potato dextrose agar, Conda Laboratories, Spain), Rose Bengal-chloramphenicol agar (Conda Laboratories, Spain), malt extract agar (MEA; malt extract 2%, peptone 0.6%, dextrose 2%, agar 1.7%), and GYPA (1% dextrose, 0.5% peptone, 0.5% yeast extract, 2.5% NaCl, 2% agar). Chloramphenicol (0.05%) was added to all media, excluding Rose Bengal agar, which already contained this antibiotic. Media were prepared using either 50% filtered seawater or supplemented with 1.5% NaCl, except for GYPA, which was prepared without these modifications. Inoculated plates were incubated at 25 °C, and colony growth was monitored over a period of 3–7 days, with additional inspection when necessary to confirm the absence of new distinguishable yeast colonies. To complement the filtration procedure and enhance the recovery of viable yeasts potentially present at low concentrations, an enrichment protocol adapted from Zaky et al. (2016) was also applied, particularly for sediment samples. Colonies from each seawater and sediment sample collected at each site and season were grouped into morphotypes based on macroscopic and microscopic characteristics (color, size, shape, surface texture, and margin). A stratified morphotype-based selection was performed, whereby 1–2 colonies were selected from less-represented morphotypes and 3–5 colonies from more-represented morphotypes, reducing redundancy while capturing the breadth of culturable diversity (Fotedar et al. 2022; Xue et al. 2024). This strategy retained a semi-quantitative component by preserving the relative representation of morphotypes as proxies for taxa within the culturable assemblage. Purified isolates were preserved in YPD broth supplemented with 25% (v/v) glycerol at –80 °C for later identification.

### Molecular identification and phylogenetic analysis

DNA was extracted from pure cultures of the isolated yeasts using the MasterPure™ Yeast DNA Purification Kit (Fisher Scientific), according to the manufacturer’s instructions. Identification was performed by amplification of the internal transcribed spacer (ITS) region, which includes the 5.8S rRNA gene, and the D1/D2 domain of the large subunit (LSU) rRNA gene by PCR, using universal primers ITS1 (5’-TCC GTA GGT GAA CCT GCG G-3’) and ITS4 (5’-TCC TCC GCT TAT TGA TAT GC-3’) or primers NL1 (5’-CAT ATC AAT AAG CGG AGG AAA AG-3’) and NL4 (5’-GGT CCG TGT TTC AAG ACG G-3’), respectively (Kurtzman and Robnett 1998; Kurtzman 2014). PCR products were verified by agarose gel electrophoresis and purified with the GeneJET PCR Purification Kit (Fisher Scientific). The purified products were submitted to the laboratories of the Molecular Biology Service of the SAI (Research Support Service, University of Murcia) for sequencing, using the same primers as those employed for PCR amplification. The resulting sequences were analyzed using NCBI-BLAST (http://www.ncbi.nlm.nih.gov/BLAST/) and compared against those of type strains available in reference databases for species identification (Fig. 1B). To support species-level taxonomic assignment, sequences were grouped using pragmatic similarity criteria of ≥ 98.4% for ITS and ≥ 99.5% for LSU D1/D2, based on previous studies reporting these ranges as useful for reliable identification of closely related yeast species (Vu et al. 2016; reviewed in Boekhout et al. 2021). Species-level assignments were accepted when either ITS or LSU sequence similarity met the corresponding operational similarity criterion and was consistent with phylogenetic placement. Isolates with sequence similarity below these criteria were conservatively assigned at the genus level and considered to represent “potentially novel species,” for which further study is needed before formal description. For some closely related taxa, particularly within the genera *Clavispora* and *Metschnikowia*, species-level resolution based solely on ribosomal markers may be limited due to high sequence heterogeneity, and identifications were therefore interpreted with caution (Vu et al. 2016; reviewed in Boekhout et al. 2021). For phylogenetic reconstruction, ITS sequences of representative strains were used. These sequences were aligned using the MUSCLE algorithm. The final alignment comprised 1,329 positions, including 722 parsimony-informative sites. To accommodate variation in sequence length and the presence of gaps among taxa, a pairwise deletion approach was applied to maximize retention of phylogenetic information. Phylogenetic trees were inferred in MEGA11 (Tamura et al. 2021) with the Neighbor-Joining method under the Kimura 2-parameter model. Evolutionary distances were estimated under this model, assuming uniform substitution rates and incorporating both transitions and transversions. The robustness of the tree topology was tested by 1,000 bootstrap replicates, considering clades with strong support as reliable (Lachance 2022).

### Phenotypic and stress tolerance analysis

Phenotypic and stress tolerance analyses of representative isolated yeast strains were performed on YPD agar plates supplemented with NaCl (0%, 5%, 10%, or 15% w/v) or freshly prepared H_2_O_2_ (1 mM, 5 mM, or 10 mM) to evaluate osmotolerance and oxidative stress resistance, respectively. Briefly, strains were precultured in YPD broth for 48 h at 25 °C with shaking at 150 rpm. The optical density (OD_600_) was measured, and cell suspensions were adjusted to an OD of 0.5. Subsequently, 10 µL aliquots were spotted onto the indicated YPD agar plates and incubated for 5 days at 25 °C. To evaluate thermotolerance, YPD plates were incubated at different temperatures (the range tested was from 10 °C to 37 °C) for 5 days.

### Microscopy

Yeast cells were imaged using a Leica THUNDER widefield microscope equipped with differential interference contrast (DIC) optics. Yeast strains were cultured in YPD broth medium at 25 °C to mid-log phase. Cells were harvested, washed with phosphate-buffered saline (PBS), and immobilized on poly-L-lysine-coated coverslips. Images were acquired using a 63×/1.4 NA oil immersion objective under consistent DIC settings. Image analysis was performed with Leica LAS X and Fiji/ImageJ software.

### Enzymatic characterization of representative strains

Extracellular enzyme production by representative yeast isolates was characterized using two complementary approaches (Fig. 1B). First, general enzymatic profiles were evaluated with the API-ZYM system (BioMérieux, France), a semi-quantitative micromethod designed to assess 19 enzymatic activities. Additionally, representative strains were screened for lytic enzymes of industrial relevance using semi-quantitative plate assays. All assays were carried out using the same volume of medium per plate on yeast malt agar (YM; 0.3% yeast extract, 0.3% malt extract, 0.5% peptone, 1% glucose, 2% agar) and the appropriate substrate for enzyme activity, as previously described (Carrasco et al. 2012; Martorell et al. 2017). Briefly, six lytic activities were tested: amylase (YM containing 0.2% soluble starch), protease (YM supplemented with 1% skim milk), xylanase (YM containing 0.5% xylan), pectinase (YM containing 1% pectin), cellulase (YM containing 0.5% carboxymethylcellulose), and chitinase (YM containing 2.5% purified chitin). Strains were precultured in YM broth for 48 h at 25 °C with shaking at 150 rpm. Cell suspensions were adjusted to an OD_600_ of 1. Subsequently, 10 µL aliquots were spotted onto the indicated plates and incubated for 5 days at 25 °C. Exoenzymatic activity was quantified in all cases by measuring the diameter of the halo (in mm) around the colony, corresponding to either coloration or discoloration after flooding the plates with the appropriate reagent (Martorell et al. 2017).

### Statistical analysis

All metrics were derived from taxonomically identified yeast isolates obtained through the culture-dependent and morphotype-based procedures described above and, therefore, refer to the culturable yeast assemblages recovered under the applied methodology. Throughout this study, “abundance” refers to isolate-based abundance, defined as the number of identified isolates of each taxon within the analytical unit considered. Relative abundance was calculated as the proportion of isolates of each taxon relative to the total number of identified isolates within each substrate, site or season, depending on the analysis. Frequency of occurrence was defined as the proportion of samples in which each taxon was detected, based on presence–absence data. To capture different ecological dimensions, two complementary species-level data frameworks were used: (i) an abundance matrix based on isolate counts for abundance-based diversity analyses and Bray–Curtis dissimilarities and (ii) a presence–absence matrix for incidence-based analyses using Jaccard distances. Substrate type, sampling zone, and season were included as sample-level metadata and used as grouping factors. All statistical analyses were performed in R v4.4.3 (R Core Team, 2025), and diversity metrics were calculated using the *vegan* package v2.6-10 (Oksanen et al. 2025).

Yeast species diversity was assessed at different ecological scales using alpha, beta, and gamma diversity analyses. Gamma diversity was estimated by pooling species-level isolate counts within each substrate category (seawater and sediment) and quantified using observed richness (S_obs_), Shannon diversity (H'), and the Chao1 richness estimator. Alpha diversity was analyzed across spatial and temporal scales using observed richness (S_obs_), Shannon diversity (H'), Simpson diversity (1 – *D*), Margalef’s richness index, and the Chao1 richness estimator. Spatial patterns were assessed using cumulative alpha diversity per site, calculated by combining seasonal samples within each habitat, and integrated alpha diversity per zone and season, calculated by combining seawater and sediment samples. Temporal variation was evaluated using sites as replicates within each habitat and within the integrated water–sediment dataset. Diversity indices were calculated from the isolate-based abundance matrix using functions from the *vegan* package in R, with Margalef’s richness index computed according to the formula:$D_{\mathrm{Mg}}=\frac{S_{\mathrm{obs}}-1}{\ln N}$ , where S_obs_ is the observed number of species and *N* is the total number of identified isolates in the corresponding sample or grouping level. Statistical differences were tested using one-way ANOVA after verifying independence of observations, normality (Shapiro–Wilk test), and homogeneity of variances (Levene’s test). *Post hoc* comparisons were adjusted using the false discovery rate (FDR).

Beta diversity was assessed using two complementary approaches: (i) Jaccard distance (1 – Jaccard similarity), calculated from presence–absence data, to evaluate taxonomic differences between geographic zones and among seasons and (ii) Bray–Curtis dissimilarity, calculated from square-root-transformed abundance data. Total beta diversity was partitioned into turnover (β_sim_) and nestedness (β_sne_) components based on Sørensen dissimilarity (β_sor_) using the betapart R package v1.6.1 (Baselga et al. 2025). Community variation was visualized using non-metric multidimensional scaling (NMDS) based on Bray–Curtis dissimilarities. Differences between groups were tested using permutational multivariate analysis of variance (PERMANOVA; 999 permutations), following verification of homogeneity of multivariate dispersions (*betadisper*). SIMPER analysis was performed on the Bray–Curtis dissimilarity matrix to identify the taxa contributing most to between-group dissimilarities.

To explore environmental gradients and associations among physicochemical variables across sampling areas, principal component analysis (PCA) was performed on normalized environmental data implemented using the *FactoMineR* package v2.11 (Lê et al. 2008). The influence of environmental variables on community composition was assessed using PERMANOVA (999 permutations) based on Bray–Curtis dissimilarities. Both global models, including all selected predictors, and marginal tests evaluating the individual contribution of each variable were performed. In parallel, redundancy analysis (RDA) was conducted on Hellinger-transformed abundance data to visualize community–environment relationships. Prior to RDA, environmental variables were screened for multicollinearity using variance inflation factors (VIF < 5). The significance of the RDA model was evaluated using permutation ANOVA.

Data visualization was performed using the *ggplot2* package (Wickham 2016) and GraphPad Prism v9. Statistical significance was defined as follows: ns = *p* > 0.05; *p < 0.05; **p < 0.01; and ***p < 0.001.

## Results

### Marine physicochemical parameters relevant to yeast assemblages

Seawater and sediment samples were collected from ten coastal sites along the Murcian Mediterranean coast between autumn 2024 and summer 2025 to characterize the physicochemical context associated with culturable yeasts (Fig. 1). Water column parameters displayed moderate seasonal variability consistent with Mediterranean conditions (Table 1). Surface temperature ranged from 13 °C to 31 °C, averaging ~15.5 °C in winter and ~29.8 °C in summer. Dissolved oxygen showed an inverse pattern, from 7.5 mg L^–1^ in winter to 6.3 mg L^–1^ in summer, while pH remained stable within a slightly alkaline range (8.08–8.15). Salinity, conductivity, and total dissolved solids (TDS) exhibited minimal seasonal variation and were strongly correlated, indicating stable ionic composition across most sites. The Mar Menor lagoon (site 9), included in the calculation of mean values, showed relatively high values, with maximum recorded values of 41.8 g L^–1^ (salinity), 59.4 mS cm^–1^ (SPC), and 42.5 g L^–1^ (TDS) at this site. As a coastal lagoon with distinct physicochemical characteristics, its inclusion improves the representation of the environmental variability of the region’s waters. In contrast, the Salt Ponds of San Pedro del Pinatar (site 10) markedly diverged, with extreme salinity (70.4 g L^–1^), conductivity (104.8 mS cm^–1^), and TDS (74.4 g L^–1^), reflecting active salt exploitation and generating a high-osmotic niche likely favoring extremotolerant yeast taxa.

**Table 1.** Seasonal variation of physicochemical water parameters*.

Study Season	pH	Temperature (°C)	DO (mg L^-1^)	Salinity (g L^-1^)	SPC (mS cm^-1^)	TDS (g L^-1^)
Autumn	8.08 ± 0.05	23.09 ± 0.60	6.90 ± 0.50	37.20 ± 2.90	52.30 ± 2.70	37.30 ± 1.90
Winter	8.11 ± 0.08	15.50 ± 0.50	7.50 ± 0.50	37.80 ± 1.80	53.30 ± 2.10	37.90 ± 1.70
Spring	8.15 ± 0.10	18.70 ± 1.12	7.10 ± 1.10	37.19 ± 0.60	52.80 ± 0.90	37.60 ± 0.60
Summer	8.09 ± 0.03	29.80 ± 0.95	6.30 ± 0.90	36.30 ± 2.40	50.30 ± 1.04	35.90 ± 0.70

(*) Mean ± SD of physicochemical water parameters measured at sampling sites 1–9 across four seasons (autumn 2024 to summer 2025). Data from the Salt Ponds (site 10), reflecting their extreme environmental conditions, were excluded from the averages and are discussed in the text. DO, dissolved oxygen; SPC, specific conductance; TDS, total dissolved solids.

**Figure 2.**
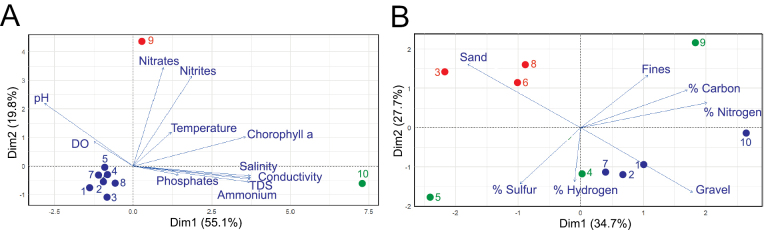
Principal component analysis (PCA) of physicochemical parameters at sampling sites. Correlation biplots showing the relationships among physicochemical parameters of water **A** and sediment **B** and their associations with the sampling sites (1–10). All values represent the mean of measurements from ten sampling zones across the four seasons. Colors represent the distinct clusters identified through the multivariate analysis. DO, dissolved oxygen; TDS, total dissolved solids.

Principal component analysis (PCA) of water parameters indicated clear spatial heterogeneity among sampling sites (Fig. 2A). The Mar Menor sampling area (site 9) clustered separately due to elevated nitrate, nitrite, and chlorophyll-a, consistent with nutrient enrichment from agricultural runoff (Pérez-Ruzafa et al. 2019). The Salt Ponds (site 10) also diverged strongly, defined by extreme salinity, conductivity, TDS, and elevated ammonium. In contrast, the remaining open-coastal sites grouped more closely, reflecting stable conditions typical of less-impacted Mediterranean waters. Sediment analyses provided complementary evidence of benthic divergence across sites (Fig. 2B). Elevated carbon and nitrogen contents confirmed organic enrichment under eutrophic conditions in the Mar Menor sampling area (site 9). In the Port of Cartagena (site 4), high hydrogen concentrations were consistent with organic matter accumulation in a poorly flushed harbor basin. At Portmán (site 5), sulfur enrichment reflected the legacy of historical mining discharges from the adjacent petrochemical complex (Cesar et al. 2009). Granulometric variation further distinguished sites: sandy substrates at La Manga (site 8), Calblanque (site 6), La Azohía (site 3), and Mar Menor (site 9) contrasted with coarser gravel- and pebble-dominated sediments at Cabo de Palos (site 7), Port of Águilas (site 1), and Calnegre (site 2). Rocky and coarse sediments (>2.5 mm) are known to provide stable microhabitats that typically sustain higher microbial diversity and abundance relative to sandy environments (Kutty and Philip 2008).

Overall, the combination of moderate seasonal dynamics (Table 1), site-specific contrasts in water column parameters (Fig. 2A), and distinct sedimentary profiles (Fig. 2B) delineates a heterogeneous physicochemical setting. This environmental mosaic establishes the abiotic framework governing yeast occurrence and diversity, forming the basis for subsequent ecological analyses.

### Overview of the taxonomic and phylogenetic diversity of culturable marine yeasts

Yeasts were successfully isolated and cultivated from all sampling sites, encompassing both water and sediment samples, throughout all four seasons. Given that yeast densities in marine environments are typically low (Fell 2001; Kutty and Philip 2008), two complementary cultivation strategies were employed, as described in the Materials and methods, to maximize the recovery of culturable yeast diversity across different culture media. GYPA medium showed a qualitative tendency toward higher colony recovery than the other media under the applied culture conditions in both water and sediment samples. Sequence analysis of the ITS region or the D1/D2 domain identified 415 strains, taxonomically assigned to 44 genera and 90 species proxies, grouped according to sequence similarity values (Suppl. material 1). Twelve isolates represent potential novel species, nine of which belong to the phylum *Ascomycota* and three to *Basidiomycota* (Suppl. material 1), based on BLAST sequence identity values below the operational similarity criteria adopted in this study to obtain preliminary but reliable species-level taxonomic assignments (Vu et al. 2016; Boekhout et al. 2021).

**Figure 3.**
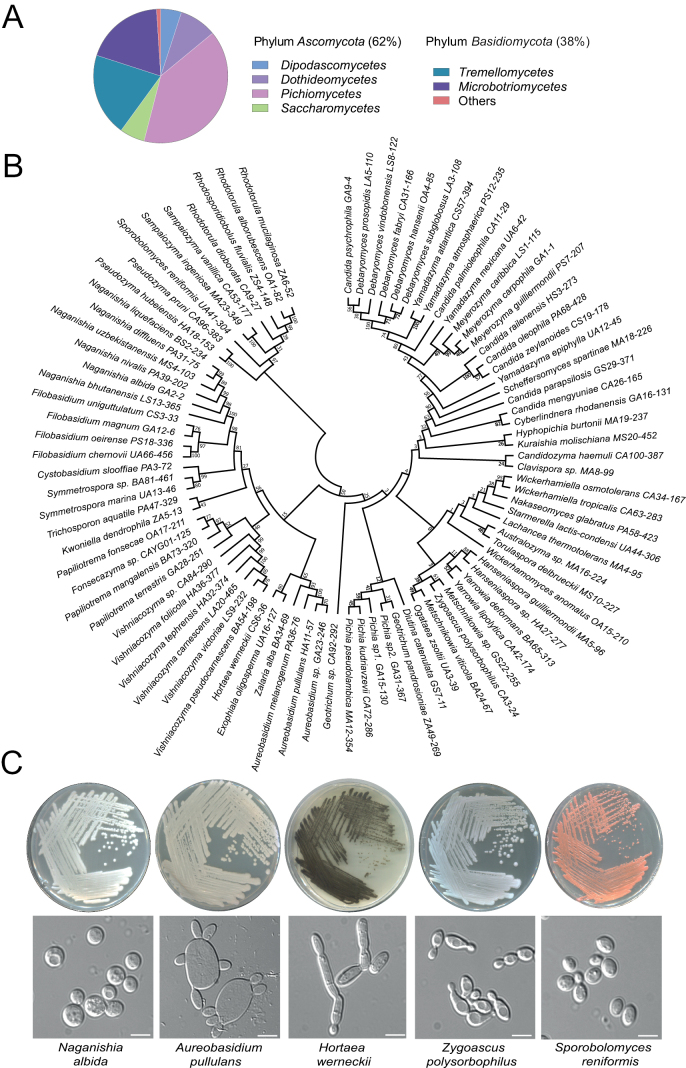
Taxonomic and phylogenetic diversity of culturable marine yeasts. **A** Taxonomic distribution of isolates at the phylum and class levels. **B** Neighbor-Joining phylogenetic tree based on ITS sequences. **C** Colony morphology and pigmentation on GYPA medium; DIC images of exponentially growing liquid cultures are shown below. Scale bar: 10 μm.

Overall, 62% of the identified species (56 species across 30 genera) were assigned to *Ascomycota*, primarily distributed among the classes *Pichiomycetes*, *Dothideomycetes*, *Saccharomycetes*, and *Dipodascomycetes*. The remaining 38% (34 species across 14 genera) were affiliated with *Basidiomycota*, mainly comprising the classes *Tremellomycetes* and *Microbotryomycetes* (Fig. 3A). An overview of the taxonomic distribution of the isolates is shown in Fig. 3B. However, this arrangement does not necessarily represent true phylogenetic relationships, as several yeast genera (e.g., *Candida*) are known to be polyphyletic and dispersed across different fungal lineages (Kurtzman and Robnett 1998; Xue et al. 2024). Approximately 10% of the isolates corresponded to stress-tolerant black yeast-like fungi (*Aureobasidium* spp., *Hortaea werneckii*, *Exophiala oligosperma*, and *Zalaria alba*), while red-pigmented taxa (*Rhodotorula*, *Cystobasidium*, *Sporobolomyces*, *Symmetrospora*, and *Rhodosporidiobolus*) were also abundant (Suppl. material 1, Fig. 3B). The high yeast diversity observed was also evident from the wide range of colony morphologies and pigmentation patterns (Fig. 3C). Of particular interest is *Hortaea werneckii*, a darkly pigmented, extremely halotolerant species identified at multiple locations, remarkable for its ability to divide by both fission and budding (Mitchison-Field et al. 2019; Zalar et al. 2019). Similarly, *Aureobasidium pullulans*, a widely distributed black yeast-like fungus of biotechnological relevance, forms pale pink colonies that gradually melanize and produces multiple simultaneous buds per cell cycle (Rekha and Sharma 2007; Mitchison-Field et al. 2019).

### Habitat-specific distribution of marine yeasts in water and sediment

The distribution and diversity of marine yeasts are strongly shaped by environmental factors that define their ecological niches and survival strategies (Kutty and Philip 2008; Cogliati et al. 2023; Lobo et al. 2024). The distribution and composition of culturable yeast assemblages differed markedly between the water column and benthic sediments. A total of 68% (*n* = 285) of the isolated yeast strains were obtained from seawater samples, while 32% (*n* = 130) originated from sediments.

**Figure 4.**
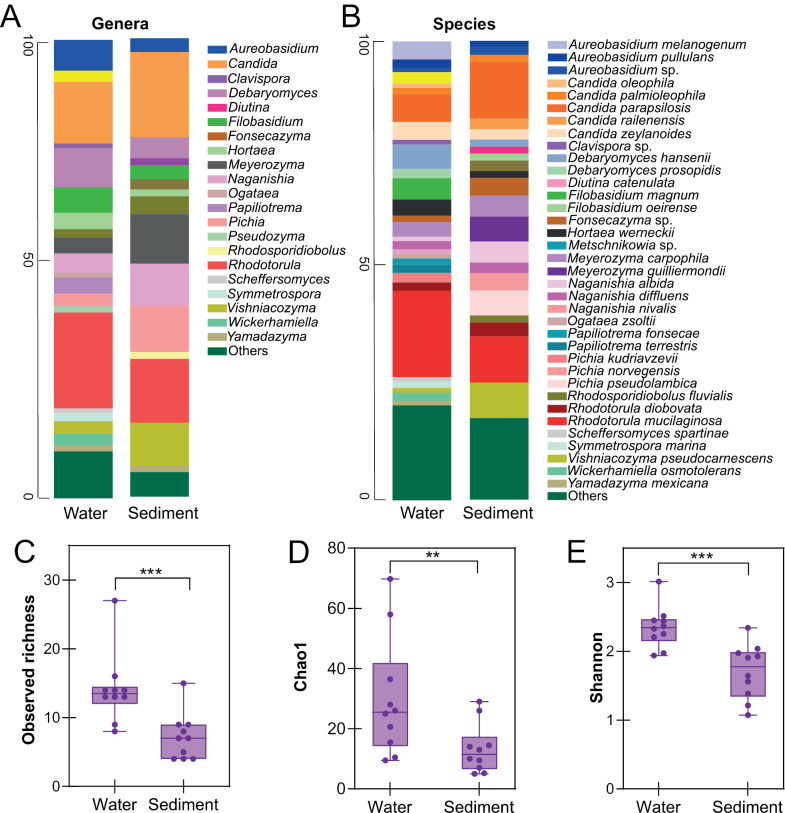
Taxonomic composition and gamma diversity of culturable yeast isolates from seawater and sediment. **A** Genus-level and **B** species-level relative abundance, calculated as the proportion of identified isolates assigned to each taxon within each substrate. Gamma diversity was calculated at the species level by pooling all seawater samples from the ten sampling zones across the four seasons and, separately, all sediment samples. Panels show observed richness **C** Chao1 estimated richness **D** and Shannon diversity **E** for seawater and sediment. Statistical significance was set as follows: ns = *p* > 0.05; *p < 0.05; **p < 0.01; ***p < 0.001.

At each sampling site, equal numbers of seawater and sediment samples were collected and processed for yeast isolation, ensuring a balanced sampling design for both substrates, with yeast colonies recovered from all samples. Among the 44 identified genera, 41 were detected in seawater and 22 in sediment, with 19 shared between both substrates: *Aureobasidium*, *Candida*, *Cystobasidium*, *Debaryomyces*, *Diutina*, *Fonsecazyma*, *Filobasidium*, *Hortaea*, *Metschnikowia*, *Meyerozyma*, *Naganishia*, *Pichia*, *Rhodotorula*, *Vishniacozyma*, *Wickerhamiella*, *Wickerhamomyces*, *Yamadazyma*, *Yarrowia*, and *Zygoascus* (Fig. 4A). Only three genera were restricted to sediment (*Kuraishia*, *Rhodosporidiobolus*, and *Torulaspora*), whereas 22 were detected exclusively in seawater, including *Australozyma*, *Candidozyma*, *Clavispora*, *Cyberlindnera*, *Exophiala*, *Geotrichum*, *Hanseniaspora*, *Hyphopichia*, *Kwoniella*, *Lachancea*, *Nakaseomyces*, *Ogataea*, *Papiliotrema*, *Pseudozyma*, *Sampaiozyma*, *Saturnispora*, *Scheffersomyces*, *Sporobolomyces*, *Starmerella*, *Symmetrospora*, *Trichosporon*, and *Zalaria* (Fig. 4A). The genera with the highest relative abundance in both substrates, calculated separately for seawater and sediment samples based on identified isolates, were *Rhodotorula* (19% in seawater, 13% in sediment) and *Candida* (17% in sediment, 13% in seawater). Other taxa with high relative abundance in the isolate dataset included *Meyerozyma* (12% in sediment, 5% in seawater), *Vishniacozyma* (10% in sediment, 3% in seawater), *Debaryomyces* (9% in seawater, 5% in sediment), *Pichia* (9% in sediment, 3% in seawater), *Naganishia* (8% in sediment, 4% in water), and *Aureobasidium* (6% in seawater, 1% in sediment) (Fig. 4A).

At the species level, of the 90 yeast species isolated, 77 were detected in seawater and 44 in sediment. Among these, 31 species were shared between both habitats, whereas 46 were exclusive to seawater and 13 were unique to sediment. The two species with the highest relative abundance in both environments were *Rhodotorula mucilaginosa* (25% in water and 10% in sediment) and *Candida parapsilosis* (8% in water and 17% in sediment) (Fig. 4B).

To assess overall species richness and diversity within each habitat, gamma diversity was estimated separately for seawater and sediment by pooling species-level isolate counts across all sites and seasons. Observed richness, Chao1 estimated richness, and Shannon diversity were then calculated for both substrates. The analysis revealed significantly higher species richness and diversity in seawater than in sediment (Fig. 4C–E), in agreement with recent large-scale studies in marine environments (Zhu et al. 2023). High yeast diversity in the water column may result from increased dispersal and nutrient availability, while sediments impose selective pressures that limit species, with some marine yeasts adapted to survive in low-oxygen conditions (Bass et al. 2007; Fell 2012).

### Spatial distribution of culturable marine yeast communities across sampling locations

**Figure 5.**
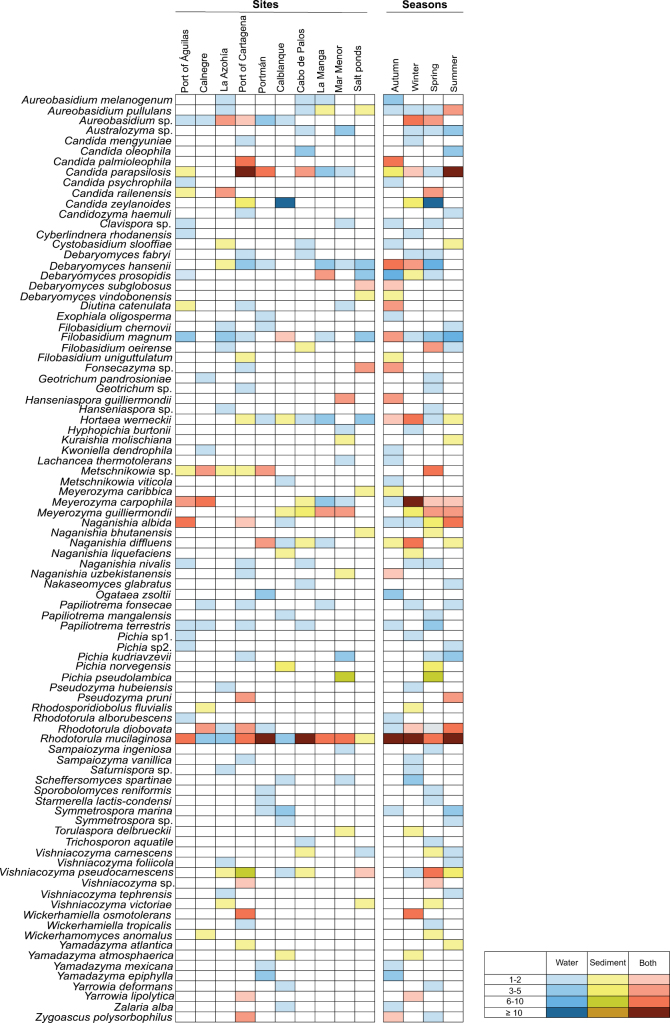
Culturable yeast species richness by site and season. Colored and white squares indicate, respectively, the presence and absence of each species based on identified isolates, reflecting their frequency of occurrence across sites and seasons. Color indicates substrate origin: seawater (blue), sediment (yellow), or both substrates (red). Color intensity represents isolate-based abundance, defined as the number of identified isolates assigned to each species within each site/season category.

Beyond substrate-related differences, the spatial distribution of yeast communities across marine zones highlights how environmental and anthropogenic pressures shape community composition and abundance (Kutty and Philip 2008). The results revealed site-specific variation in the composition and diversity of culturable yeast assemblages across the study area, as further detailed below through taxonomic composition, alpha-diversity indices, and beta-diversity analyses. Only *Rhodotorula mucilaginosa* was detected in all ten sampling sites, either in seawater or sediment, showing the highest frequency of occurrence across the Mediterranean coastal gradient (Fig. 5). Taxa showing both high frequency of occurrence, defined as recovery from at least 80% of the zones, and high isolate-based abundance were *Rhodotorula* (72 isolates from three species, *Rhodotorula alborubescens*, *Rhodotorula diobovata*, and *Rhodotorula mucilaginosa*); *Candida* (63 isolates from nine species, with *Candida parapsilosis* most frequent); *Debaryomyces* (31 isolates from six species, predominantly *Debaryomyces hansenii*); *Aureobasidium* (23 isolates from three species, *Aureobasidium pullulans*, *Aureobasidium melanogenum*, and a third lineage representing a potentially novel species); and *Filobasidium* (20 isolates from four species, primarily *Filobasidium magnum*) (Fig. 5).

Several site-exclusive species were also detected, each restricted to a single zone and sample type (water or sediment). These included *Candida psychrophila* (site 1, Port of Águilas, water); *Kwoniella dendrophila* and *Rhodosporidiobolus fluvialis* (site 2, Calnegre, water and sediment, respectively); *Vishniacozyma foliicola* and *Vishniacozyma tephrensis* (site 3, La Azohía, water); *Yamadazyma epiphylla*, *Exophiala oligosperma*, *Starmerella lactis-condensi*, and *Sporobolomyces reniformis* (site 5, Portmán, water); *Zalaria alba*, *Yarrowia deformans*, and *Papiliotrema mangalensis* (site 6, Calblanque, water); *Naganishia liquefaciens* (site 6, sediment); *Nakaseomyces glabratus* and *Trichosporon aquatile* (site 7, Cabo de Palos, water); *Yamadazyma atmosphaerica* (site 7, sediment); *Hyphopichia burtonii* and *Sampaiozyma ingeniosa* (site 9, Mar Menor, water); and *Torulaspora delbrueckii* and *Kuraishia molischiana* (site 9, sediment). In the hypersaline site 10, halotolerant species such as *Hortaea werneckii* and various *Debaryomyces* species dominated both water and sediment samples (Fig. 5), consistent with their well-documented tolerance to high salinity (Prista et al. 1997; Zalar et al. 2019).

Most zones showed high yeast richness, a trend particularly pronounced at site 4 (Port of Cartagena), which is strongly influenced by anthropogenic activities, including commercial and tourism-related operations. Consequently, within the culture-dependent approach used, this area showed the highest richness of yeast species among the sampled sites, with 26 species recovered from the water column and 17 from sediments, including several site-specific taxa such as *Candidozyma haemuli*, *Candida mengyuniae*, and *Sampaiozyma vanillica* in water and *Filobasidium uniguttulatum* and *Yamadazyma atlantica* in sediment (Fig. 5). Although predominantly marine-associated species were detected, the human commensal *Candida parapsilosis* was the most represented species, consistent with substantial anthropogenic influence in the area (Fig. 5). Cumulative alpha diversity per habitat and zone was consistent with these findings, with the Port of Cartagena sampling point (site 4) exhibiting the highest species-level diversity and richness values across all diversity metrics: Margalef index of 6.39 (water) and 4.45 (sediment), Simpson 1 – *D* of 0.93 (water) and 0.89 (sediment), Shannon index of 2.96 (water) and 2.51 (sediment), and Chao1 index of 64.25 (water) and 34.33 (sediment) (Suppl. material 2). In other anthropogenically influenced sites, such as Mar Menor (site 9) and the Águilas fishing port (site 1), yeast assemblages also displayed elevated richness and diversity, suggesting that eutrophication and episodic anoxia may generate diverse micro-niches supporting rare or specialized taxa at low densities. In contrast, sandy sediments from Portmán (site 5), Calblanque (site 6), and La Manga (site 8) exhibited the lowest diversity indices (Suppl. material 2), consistent with the scarcity of stable surfaces suitable for colonization (Kutty and Philip 2008; Aldeguer-Riquelme et al. 2022; Giner-Lamia and Huerta-Cepas 2024).

**Figure 6.**
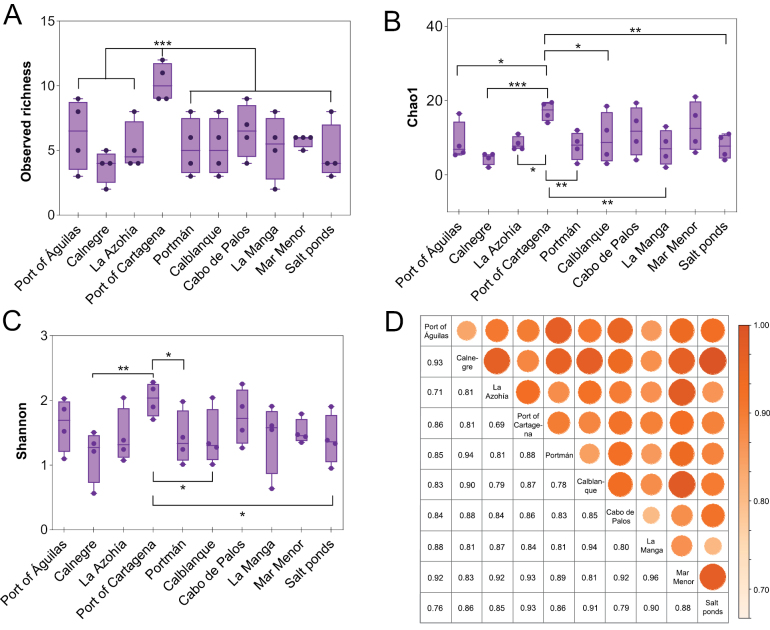
Spatial variation in alpha (richness and diversity) and beta (community dissimilarity) diversity of culturable yeast species among sampling sites. Graphical representation of observed richness **A** Chao1 estimated richness **B** Shannon diversity **C** and Jaccard distance (1 – Jaccard similarity) **D** indices per site, combining seawater and sediment data. Statistical significance was set as follows: ns = *p* > 0.05; *p < 0.05; **p < 0.01; ***p < 0.001.

To evaluate integrated species-level alpha diversity across sampling sites, seawater and sediment data were combined for each site across the four sampled seasons. Significant differences (*p* < 0.05) among sites were detected for observed richness (Fig. 6A), Chao1 estimated richness (Fig. 6B), and Shannon diversity (Fig. 6C). In all three metrics, site 4 (Port of Cartagena) exhibited the highest richness and diversity values. However, not all pairwise comparisons were significant, as some sites with elevated diversity did not differ significantly from site 4.

Beta diversity was assessed using Jaccard distance (1 – Jaccard similarity) on integrated site-level presence–absence data to evaluate variation in taxonomic composition among culturable yeast communities across coastal sites. The resulting values (0.69–0.96) indicate high dissimilarity, reflecting pronounced taxonomic differentiation between locations (Fig. 6D). To further elucidate the mechanisms driving this variation, Sørensen-based beta diversity partitioning indicated high spatial dissimilarity among sites (β_sor_ = 0.86), mainly driven by species turnover (β_sim_ = 0.81; 94.7% of total beta diversity), whereas nestedness contributed only marginally (β_sne_ = 0.04). These findings support site-specific compositional differences among culturable yeast assemblages. Overall, these results highlight the substantial diversity and spatial structuring of culturable yeast communities in this marine system and emphasize the combined influence of environmental gradients and anthropogenic pressures on microbial composition and diversity.

### Seasonal patterns in the diversity of culturable marine yeasts

The temporal variability of culturable yeast species diversity across the four meteorological seasons was further analyzed. A total of 37 species were isolated in autumn, 36 in winter, 40 in spring, and 30 in summer, suggesting that yeast richness was maintained throughout the sampling period (Fig. 5). Although some species occurred in two or three seasons, only six were consistently detected across all four in either water or sediment: *Candida parapsilosis*, *Filobasidium magnum*, *Hortaea werneckii*, *Meyerozyma carpophila*, *Naganishia albida*, and *Rhodotorula mucilaginosa*. The most represented species shifted across seasons, with *Rhodotorula mucilaginosa*, *Debaryomyces hansenii*, and *Candida palmioleophila* prevailing in autumn; *Meyerozyma carpophila*, *Rhodotorula mucilaginosa*, *Aureobasidium* sp., and *Hortaea werneckii* in winter; *Candida zeylanoides*, *Vishniacozyma pseudocarnescens*, and *Pichia pseudolambica* in spring; and *Candida parapsilosis*, in addition to *Rhodotorula mucilaginosa*, in summer. *Candida parapsilosis* showed the highest relative abundance in summer (25%), suggesting its prevalence may be linked to human presence and activity at the beaches. Several species showed seasonal and habitat-specific occurrence, suggesting dynamic shifts in marine yeast diversity alongside a stable core microbiome (Fig. 5).

Many species (8–14) were restricted to specific seasons in either water or sediment, underscoring pronounced temporal variation in marine yeast diversity (Fig. 5). *Candida palmioleophila*, *Candida psychrophila*, *Diutina catenulata*, *Exophiala oligosperma*, *Filobasidium uniguttulatum*, *Fonsecazyma* sp., *Kwoniella dendrophila*, *Lachancea thermotolerans*, *Metschnikowia viticola*, *Meyerozyma caribbica*, *Ogataea zsoltii*, *Yamadazyma epiphylla*, *Yamadazyma mexicana*, and *Zalaria alba* were exclusively isolated in the autumn. Species detected only in winter included *Candida mengyuniae*, *Cyberlindnera rhodanensis*, *Hyphopichia burtonii*, *Naganishia liquefaciens*, *Pseudozyma hubeiensis*, *Rhodosporidiobolus fluvialis*, *Sampaiozyma vanillica*, *Saturnispora* sp., *Scheffersomyces spartinae*, *Torulaspora delbrueckii*, *Yamadazyma atmosphaerica*, and *Yarrowia lipolytica*. Spring-exclusive species comprised *Geotrichum pandrosioniae*, *Naganishia bhutanensis*, *Papiliotrema mangalensis*, *Pichia pseudolambica*, *Sampaiozyma ingeniosa*, *Sporobolomyces reniformis*, *Starmerella lactis-condensi*, *Trichosporon aquatile*, *Wickerhamiella tropicalis*, and *Yarrowia deformans*. Summer-restricted taxa included *Candida oleophila*, *Candidozyma haemuli*, *Filobasidium chernovii*, *Nakaseomyces glabratus*, *Pseudozyma pruni*, *Vishniacozyma foliicola*, *Vishniacozyma tephrensis*, and *Yamadazyma atlantica* (Fig. 5). Such dynamics reveal the coexistence of a stable core microbiome with season-specific taxa.

**Figure 7.**
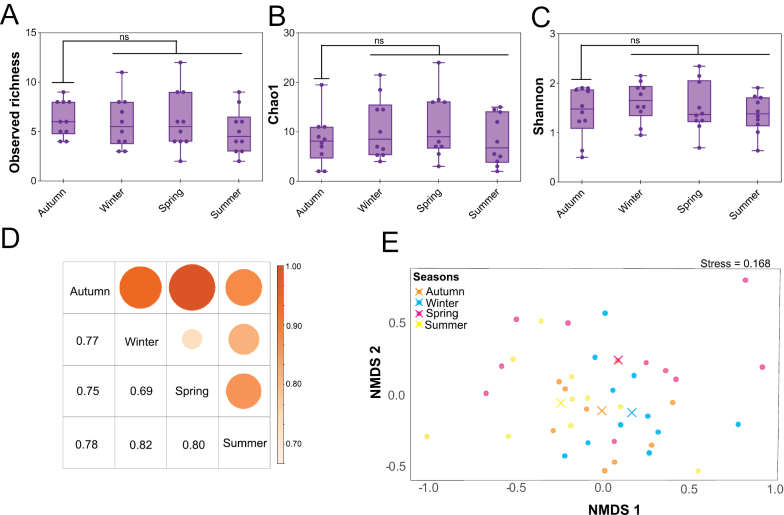
Seasonal variation in alpha (richness and diversity) and beta (community dissimilarity) diversity of culturable yeast species. Graphical representation of observed richness **A** Chao1 estimated richness **B** shannon diversity **C** and Jaccard distance (1 – Jaccard similarity) **D** indices per season, combining seawater and sediment data **E**NMDS ordination of yeast assemblages across seasons based on Bray–Curtis dissimilarities (*k* = 2; stress = 0.168). Each point represents a sampling replicate; colors indicate seasons, and crosses mark centroids. PERMANOVA showed significant seasonal differences in community composition (*F* = 1.502, *p* = 0.003). Statistical significance was set as follows: ns = *p* > 0.05.

To evaluate seasonal patterns, cumulative alpha diversity was calculated separately for seawater and sediment by pooling species-level isolate counts across sampling sites within each season, using the Margalef, Simpson (1 – *D*), Shannon, and Chao1 indices (Suppl. material 2). Yeast communities maintained high richness and diversity throughout the four sampled seasons, highlighting their resilience under changing environmental conditions. Diversity indices exhibited a seasonal trend, with reduced richness and diversity observed during summer, particularly in sediment samples (Margalef = 2.94; Simpson 1 – *D* = 0.83; Shannon = 2.06; and Chao1 = 14.33). In contrast, higher diversity values were recorded during spring, autumn, and the mild winter period, likely associated with more moderate temperatures. Specifically, diversity indices peaked in spring seawater (Margalef = 7.35; Simpson 1 – *D* = 0.94; and Shannon = 3.13), while species richness reached its highest value in autumn (Chao1 = 109) (Suppl. material 2). Analyses of integrated species-level alpha diversity, obtained by combining seawater and sediment data, supported the hypothesis of relatively stable trends of culturable yeast diversity across the four sampled seasons. Observed richness (Fig. 7A), Chao1 estimated richness (Fig. 7B), and Shannon diversity (Fig. 7C) showed consistent patterns among sites, suggesting that overall community composition remained relatively constant despite seasonal fluctuations. Although differences were not statistically significant, both species richness and diversity tended to increase during the mild Mediterranean seasons compared with summer. This seasonal pattern is partially supported by the multivariate analysis of environmental drivers (Suppl. material 4). The global PERMANOVA model revealed a significant relationship between physicochemical parameters and community structure (*R*^2^ = 0.165, *p* = 0.007), with temperature as the only significant individual factor (*p* = 0.045), accounting for only 13.6% of the variance. These results suggest that, although thermal gradients reflect seasonal shifts, other unmeasured ecological processes and influxes from external sources may additionally contribute to the structuring of yeast assemblages.

While alpha diversity remained relatively constant, Jaccard distance values (0.69–0.82) revealed strong seasonal shifts in yeast community composition, with few species shared across seasons (Fig. 7D). PERMANOVA confirmed that these compositional changes were statistically significant, indicating that the overall structure of yeast assemblages differed between seasons (*p* < 0.01). Non-metric multidimensional scaling (NMDS) ordination provided a clear visualization of this ecological gradient (Fig. 7E). SIMPER analysis further identified the species contributing most to these dissimilarities, highlighting both core taxa and season-specific species driving the observed community shifts (Suppl. material 3). These results reveal the dynamic composition of culturable marine yeast communities along the Mediterranean coast and their sensitivity to fluctuating environmental and physicochemical factors.

### Functional characterization of representative marine yeast isolates: stress tolerance and biotechnological relevance

Marine yeasts exhibit notable environmental resilience and metabolic versatility, producing diverse bioactive compounds and enzymes that highlight both their ecological significance and their potential for biotechnological applications (Zaky et al. 2014; Sarkar and Rao 2016). Physiological and enzymatic assays were performed on the 90 representative strains listed in Suppl. material 1 to assess their environmental stress tolerance and biotechnological potential. Temperature tolerance assays (10–37 °C) showed that most isolates (60%) grew between 10 °C and 25 °C, with optimal growth at 18–25 °C. A minor fraction of species (15%), including *Aureobasidium melanogenum*, *Geotrichum pandrosioniae*, *Hanseniaspora guilliermondii*, *Trichosporon aquatile*, and *Zalaria alba*, tolerated the full range of temperatures. Salt tolerance assays (5%, 10%, and 15% NaCl) revealed that all isolates grew at 5% NaCl, and several isolated species (⁓ 40%) showed tolerance to 10–15% NaCl, including *Aureobasidium melanogenum*, *Aureobasidium pullulans*, *Candidozyma haemuli*, *Filobasidium uniguttulatum*, *Hanseniaspora guilliermondii*, *Hortaea werneckii*, *Hyphopichia burtonii*, *Lachancea thermotolerans*, *Rhodotorula mucilaginosa*, and *Yamadazyma mexicana*, among others. Most of the tested yeasts (~90%) exhibited resistance to oxidative stress induced by 1 mM H_2_O_2_, whereas the melanized species *Aureobasidium pullulans* and *Aureobasidium melanogenum* (5 mM), as well as *Hortaea werneckii* and *Rhodotorula mucilaginosa* (≥2 mM), showed higher tolerance to this compound (Table 2, Suppl. material 5). These physiological traits align with previously reported marine yeast adaptations, such as high salinity tolerance (*Hortaea werneckii*, *Aureobasidium* spp.) and pigment-mediated oxidative protection (*Rhodotorula* spp.) (Prista et al. 1997; Gostinčar and Gunde-Cimerman 2024; Ochoa-Viñals et al. 2024).

**Table 2.** Exoenzymatic activities and stress tolerance in representative yeast strains with at least one positive activity.

Representative yeast species	Code	Enzyme activities halo (mm)*						Some physiological characteristics and stress tolerance**		
		Ami	Cel	Pec	Xyl	Chi	Pro	Temp. (°C)	NaCl (%)	H_2_O_2_ (mM)
*Aureobasidium melanogenum*	PA36-76	2	6	3	9	5	2	10–37	15	5
*Aureobasidium pullulans*	HA11-57	4	12	7	9	6	10	10–25	15	5
*Aureobasidium* sp.	GA23-246	4	10		6		5	10–25	5	2
*Candida railenensis*	HS3-273	3						25–30	5	2
*Candidozyma haemuli*	CA100-387						5	25–30	15	2
*Filobasidium magnum*	GA12-6						4	10–25	5	<1
*Filobasidium oeirense*	PS18-336		3					25–30	5	<1
*Filobasidium uniguttulatum*	CS3-33	2		3		1		10–25	15	2
*Geotrichum pandrosioniae*	ZA49-269							10–37	5	2
*Geotrichum* sp.	CA92-292							25–30	5	<1
*Hanseniaspora guilliermondii*	MA5-96					2		10–37	15	<1
*Hortaea werneckii*	CS6-36	4		7	7	4		25–30	15	2
*Hyphopichia burtonii*	MA19-237	5		2				10–25	15	5
*Kwoniella dendrophila*	ZA5-13	2	2			1		10–25	5	1
*Lachancea thermotolerans*	MA4-95					2		10–25	15	1
*Metschnikowia viticola*	BA24-67		5				2	10–25	5	2
*Naganishia diffluens*	PA31-75							10–25	5	1
*Papiliotrema fonsecae*	OA17-211							10–25	10	5
*Papiliotrema mangalensis*	BA73-320				5			25–30	5	<1
*Papiliotrema terrestris*	GA28-251							10–25	5	5
*Pichia kudriavzevii*	CA72-286						3	25–37	5	1
*Pichia norvegensis*	BS7-326						2	25–37	5	5
*Pseudozyma hubeiensis*	HA18-153		10	3	10	5	6	10–25	5	5
*Rhodotorula diobovata*	CA3-167		2					10–25	5	2
*Rhodotorula mucilaginosa*	ZA6-52					1		10–25	15	2
*Sampaiozyma ingeniosa*	MA23-349	10		7	10		6	25–30	5	2
*Sampaiozyma vanillica*	CA53-177	14		9		15	4	25–30	10	1
*Sporobolomyces reniformis*	UA41-304							25–30	5	2
*Symmetrospora marina*	UA13-46	8		3	6	5		25–30	5	1
*Trichosporon aquatile*	PA47-329	2	7					10–37	10	2
*Yamadazyma epiphylla*	UA12-45							10–25	10	<1
*Yamadazyma mexicana*	UA6-42	4	7		3	10		25–30	15	2
*Yarrowia deformans*	BA65-313	5					4	10–25	5	2
*Yarrowia lipolytica*	CA42-174						2	10–25	10	1
*Zalaria alba*	BA34-69	5	4		9			10–37	5	2

(*) Halo diameter around the colony (mm), determined from the colony edge to the outer halo limit: Ami, amylase; Cel, cellulase; Pec, pectinase; Xyl, xylanase; Chi, chitinase; Pro, protease. (**) Values correspond to the growth temperature range and the maximum tolerance for the indicated salt and hydrogen peroxide concentrations.

Six extracellular enzyme activities were tested (amylase, cellulase, pectinase, xylanase, chitinase, and protease), and approximately 40% of the representative strains showed at least one positive reaction, with most exhibiting multiple enzymatic activities (Table 2). *Symmetrospora marina* and *Sampaiozyma vanillica* demonstrated notable amylase and chitinase activities, while the melanized yeast *Aureobasidium pullulans* was positive for all six enzymes (Table 2). Beyond this species, black yeast-like fungi represented 10% of the total identified isolates and comprised six species, with five (*Aureobasidium* spp., *Hortaea werneckii*, and *Zalaria alba*) displaying three or more of the enzymatic activities tested (Table 2). Overall, this group accounted for approximately 15% of the enzymatically active representative strains, highlighting its contribution to the enzymatic potential detected among the culturable yeasts recovered from Mediterranean coastal environments.

To expand the enzymatic profiles of the isolates, the standardized API-ZYM system (BioMérieux) was applied to selected strains, assessing 19 activities beyond those tested on agar plates. All strains expressed at least five activities, most frequently esterase C4, esterase-lipase C8, leucine arylamidase, acid phosphatase, and naphthol-AS-BI-phosphohydrolase. Several species, including *Aureobasidium pullulans*, *Aureobasidium melanogenum*, *Hortaea werneckii*, *Zalaria alba*, and *Sampaiozyma vanillica*, exhibited more than 50% positive activities, underscoring their potential as biotechnological resources (Suppl. material 6). Additionally, some of these yeasts, recognized as opportunistic pathogens in human-impacted areas, may serve as bioindicators of marine contamination (Hagler 2006; Cogliati et al. 2023). Overall, the results underscore their dual ecological and applied significance, reflecting both stress tolerance and biotechnological potential alongside clinical relevance.

## Discussion

Marine yeasts represent an underexplored yet ecologically and biotechnologically relevant component of marine microbiomes, contributing to organic matter degradation, nutrient cycling, and microbial interactions (Kutty and Philip 2008; Zaky et al. 2014). Nevertheless, spatiotemporal studies integrating both water- and sediment-associated communities in Mediterranean coastal systems remain scarce. This study provides the first integrative overview of culturable marine yeasts along the Murcian Mediterranean coast, revealing spatial and seasonal patterns shaped by environmental heterogeneity and highlighting their biotechnological potential as stress-tolerant microorganisms.

A total of 415 yeast isolates were recovered in this study, representing 44 genera and 90 species, including 12 putative novel taxa, with 62% of the identified species belonging to *Ascomycota* and 38% to *Basidiomycota* (Fig. 3, Suppl. material 1). Although culture-dependent methods typically underestimate total richness (Kutty and Philip 2008), they are effective for recovering rare taxa for functional tests and bioprospecting studies. A broader comparison of the isolates with published culture-based surveys from other marine regions (Pacific, Atlantic, Indian, and Southern Oceans) indicates that approximately 60% of the species recovered in this study have been previously reported elsewhere, highlighting the cosmopolitan distribution of genera such as *Rhodotorula*, *Candida*, *Debaryomyces*, and *Meyerozyma*, which are commonly encountered in marine environments worldwide (Duarte et al. 2013; Fotedar et al. 2022; Breyer et al. 2023; Yu et al. 2023; Zhu et al. 2023; Xue et al. 2024; Matos et al. 2025). Several isolated yeasts are apparently restricted to Mediterranean coastal environments when compared with curated databases (www.marinefungi.org) and recent surveys from other locations (Jones et al. 2019; Zhu et al. 2023; Xue et al. 2024). These included *Cyberlindnera rhodanensis*, *Kuraishia molischiana*, *Kwoniella dendrophila*, *Ogataea zsoltii*, *Papiliotrema terrestris*, *Rhodosporidiobolus fluvialis*, *Starmerella lactis-condensi*, *Symmetrospora marina*, *Yamadazyma atmosphaerica*, *Yamadazyma mexicana*, *Yamadazyma epiphylla*, and *Zalaria alba* (Suppl. material 1). Notably, one of the most distinctive features of the dataset is the prevalence of pigmented taxa, particularly melanized yeast-like fungi such as *Hortaea werneckii*, consistent with previous reports from high-salinity Mediterranean environments (Zalar et al. 2019). This pattern is consistent with previous observations from hypersaline Mediterranean habitats and suggests that regional environmental conditions, especially elevated salinity, high solar irradiance, and seasonal thermal stress, may play a key role in structuring local yeast-like fungal assemblages. Moreover, the results highlight the western Mediterranean as a comparatively understudied region with respect to culturable marine yeast diversity, and the occurrence of several poorly resolved or potentially undescribed taxa is consistent with broader syntheses suggesting that warm and stress-prone environments may act as reservoirs of uncharacterized yeast diversity (Boekhout et al. 2021; Yurkov et al. 2021).

Red-pigmented taxa (*Rhodotorula*, *Cystobasidium*, *Sporobolomyces*, *Symmetrospora*, and *Rhodosporidiobolus*) and black yeast-like fungi (*Aureobasidium melanogenum*, *Aureobasidium pullulans*, *Exophiala oligosperma*, *Hortaea werneckii*, and *Zalaria alba*) were prevalent in habitats subjected to extreme environmental conditions along the Murcia coastline, including the hypersaline Mar Menor lagoon, the Salt Ponds of San Pedro del Pinatar, heavily impacted commercial and fishing ports, and recreational beaches. These sites are characterized by intense solar radiation, hypersalinity, and fluctuating moisture regimes, conditions that likely select for pigmented and melanized taxa with enhanced tolerance to UV exposure and osmotic stress. Nearly 10% of isolates were melanized yeast-like fungi, known for their slow growth and remarkable stress tolerance (Chi et al. 2009; Zalar et al. 2019; Gostinčar and Gunde-Cimerman 2024). *Hortaea werneckii*, one of the most halotolerant eukaryotes known, is a characteristic and nearly ubiquitous inhabitant of hypersaline niches such as salt ponds and solar salterns, together with other prominent halotolerant yeasts such as *Debaryomyces hansenii* (Gunde-Cimerman et al. 2009; Gostinčar and Gunde-Cimerman 2024; Ochoa-Viñals et al. 2024). These findings emphasize that the mentioned factors enable habitat-specific environmental pressure and determine the composition and distribution patterns of marine yeast communities.

The results reveal pronounced spatial heterogeneity in yeast assemblages, with the widespread occurrence of *Rhodotorula mucilaginosa* suggesting ecological plasticity and tolerance to varying physicochemical conditions. As this species is a prominent phylloplane yeast, its frequent detection in marine samples may reflect terrestrial inputs followed by survival in seawater, or the presence of stable marine-associated niches such as sediments or seagrass microhabitats (Vaksmaa et al. 2023). The higher gamma diversity observed in seawater compared with sediment suggests that the water column may act as a dynamic reservoir of culturable yeast diversity, potentially favored by greater dispersal potential and environmental variability (Figs 4, 5). This pattern aligns with large-scale surveys reporting greater species turnover in pelagic than in benthic compartments (Chang et al. 2016; Zhu et al. 2023). Within the study area, alpha diversity was particularly pronounced at the sampling point in the Port of Cartagena (site 4), one of the most important harbors in the western Mediterranean (Fig. 6, Suppl. material 2). Located in a sheltered bay directly influenced by both the open sea and the surrounding urban-industrial environment, this area is subject to intense anthropogenic pressures, including heavy maritime traffic, periodic dredging, and contamination by heavy metals, hydrocarbons, and wastewater discharges (García-Rivera et al. 2018), which have been shown to alter fungal richness and community composition in other zones (Li et al. 2023). In contrast, sandy sediments (sites 5, 6, and 8) supported lower fungal diversity, whereas rocky or muddy sediments (site 7) maintained higher richness (Suppl. material 2). These results are in line with previous observations showing that, unlike rocky or muddy substrates, sandy or unconsolidated sediments are continuously reworked by waves, leading to low organic matter content and limited stable surfaces for microbial colonization (Kutty and Philip 2008; Aldeguer-Riquelme et al. 2022; Giner-Lamia and Huerta-Cepas 2024).

Alpha diversity of marine yeast species remained relatively stable across all four seasons (Fig. 5), although clear seasonal dynamics were evident, with species richness increasing under mild temperatures (~15–25 °C, autumn to spring) and declining during the hot summer period (≥30 °C) characteristic of the semi-arid Mediterranean climate of the Murcia region (Table 1). This seasonal pattern is partially supported by the multivariate analysis of environmental drivers, which revealed that, among the environmental factors analyzed, temperature was the only significant individual factor influencing community structure (Suppl. material 4). The higher relative abundance of *Candida parapsilosis* in summer (25%) may be driven by elevated temperatures and anthropogenic inputs, potentially shaping community structure through competitive or inhibitory interactions and contributing to the observed reduction in species richness. Alternatively, such blooms could obscure the detection of other species, explaining the lower richness observed during summer. Although seasonal differences in alpha diversity were not statistically significant, beta diversity and NMDS analyses revealed marked seasonal turnover in community composition, indicating temporal structuring within marine yeast assemblages despite stable overall richness (Fig. 7). Such temporal variation is likely driven by fluctuations in environmental parameters that influence the survival and proliferation of specific taxa. Long-term monitoring across multiple years will be essential to determine whether these patterns represent recurrent seasonal dynamics or interannual variability within this Mediterranean context.

Collectively, these findings suggest that culturable marine yeast assemblages are highly diverse and shaped by spatial and seasonal gradients. Pronounced site differences and marked seasonal turnover indicate substantial compositional variability, largely driven by environmental conditions and anthropogenic influences. These patterns highlight the importance of incorporating spatially and temporally explicit approaches to better understand the processes shaping marine yeast diversity.

Beyond their ecological relevance, marine yeasts represent a largely unexploited reservoir of biological diversity with considerable biotechnological and pharmaceutical potential (Chi et al. 2016; Sarkar and Rao 2016). They show inherent tolerance to changes in salinity, temperature, pH, hydrostatic pressure, and oxidative stress. This aptitude stems from complex physiological and genetic traits, including mitogen-activated protein kinase (MAPK)-mediated signaling pathways that orchestrate global transcriptional and post-transcriptional programs that elicit structural and physiological adaptive responses (Cansado et al. 2022; de la Fuente-Colmenares et al. 2024). These traits underpin a wide range of industrial applications, as marine yeasts have been reported to produce robust enzymes, ferment in seawater, accumulate lipids, and degrade environmental pollutants (Zaky et al. 2014; Chi et al. 2016; Abaza et al. 2024). Many yeast isolates in this study exhibited remarkable tolerance to multiple stressors, including elevated salinity (10–15% NaCl) and oxidative stress (up to 5 mM H_2_O_2_) (Suppl. material 5). Black yeast-like fungi, such as *Hortaea werneckii*, thrived under hypersaline conditions and produced melanin, a strong protectant against UV and oxidative stress, while red-pigmented yeasts (*Rhodotorula*, *Cystobasidium*, *Sporobolomyces*, *Symmetrospora*, and *Rhodosporidiobolus*) synthesized carotenoids, enhancing resilience and offering industrial value (Rapoport et al. 2021; Šovljanski et al. 2025). Functional profiling highlighted the biotechnological relevance of these isolates, as many expressed multiple extracellular enzymes. A relevant example is *Aureobasidium pullulans*, a black yeast-like fungus associated with melanin production and also known for carotenoid production, which was positive for all six tested enzymatic activities, further supporting its value for applied purposes (Table 2). In line with this pattern, black yeast-like fungi accounted for approximately 15% of the enzymatically active culturable yeasts recovered in this study, suggesting that this group may represent a relevant component of the functional potential detected within the recovered assemblage. Their stress tolerance and frequent association with saline or otherwise harsh environments further support their interest as candidates for future biotechnological characterization. API-ZYM profiling confirmed broad enzymatic repertoires in *Aureobasidium pullulans*, *Aureobasidium melanogenum*, *Hortaea werneckii*, *Zalaria alba*, and *Sampaiozyma vanillica*, among others, demonstrating their suitability for industrial applications such as polysaccharide degradation, bioremediation, and bioconversion (Suppl. material 6).

The extracellular enzyme repertoire observed in these marine yeasts indicates a central role in the degradation of organic matter within coastal ecosystems, thereby contributing to carbon and nutrient recycling and influencing resource availability for other microbial community members. This functional versatility, coupled with their adaptability to coastal environmental conditions, not only demonstrates their ecological significance as key participants in coastal biogeochemical processes but also emphasizes their biotechnological potential for the degradation of complex polymers, positioning these organisms as promising candidates for both fundamental ecosystem studies and bioprospecting applications.

Marine yeasts also include opportunistic pathogens, particularly in coastal areas under strong anthropogenic influence. In this study, opportunistic species of potential clinical concern (*Candida parapsilosis*, *Exophiala oligosperma*, *Nakaseomyces glabratus*, *Diutina catenulata*, among others) were predominantly detected at impacted sites, confirming their value as bioindicators of marine contamination (Hagler 2006; Tuon and Costa 2008; van Asbeck et al. 2009; Anthonies and Vargas-Muñiz 2022; Nourrisson et al. 2023; Asogan et al. 2024; Huang et al. 2024; Nguyen et al. 2024; Xue et al. 2024; Cognialli et al. 2025; Lim et al. 2025; Rodríguez-Cerdeira et al. 2025). *Candida parapsilosis*, traditionally considered a human commensal, is increasingly detected in environmental sources and has emerged as a pathogen of clinical relevance. Its growing prevalence led the World Health Organization to designate it a high-priority fungal pathogen, highlighting the importance of linking environmental monitoring with public health surveillance (Asogan et al. 2024).

## Conclusion

This study provides the first spatiotemporal assessment of culturable marine yeast communities along the Mediterranean coast of the Region of Murcia (SE Spain), revealing unprecedented insights into their diversity, distribution, and functional potential. Yeast assemblages were strongly shaped by physicochemical gradients, habitat heterogeneity, and anthropogenic pressures, with richness peaking during mild seasons. Importantly, the isolation of stress-tolerant black yeast-like fungi, a highly resilient and biotechnologically promising group, represents a novel contribution to the regional marine mycobiome. High extracellular enzymatic activity and stress tolerance across isolates underscore their ecological role in organic matter turnover and adaptation to fluctuating conditions. Overall, marine yeasts emerge as both sensitive indicators of ecosystem health and reservoirs of robust strains with significant biotechnological potential. Future work combining cultivation-based and culture-independent approaches will be essential to fully capture their diversity, exploit their metabolic capacities, and evaluate their implications for ecosystem and public health.

## Additional information

### Artificial Intelligence (AI) use

The authors accept full responsibility for the content of the manuscript, including the disclosure of any use of AI.

No AI tools were used in the preparation of this manuscript.

### Adherence to national and international regulations

All the fungal strains used in this study have been legally obtained, respecting the Convention on Biological Diversity (Rio Convention).

### Author ORCIDs

A. Martínez-Rodrigo https://orcid.org/0009-0009-3699-0206

A. Núñez https://orcid.org/0000-0002-0781-0001

A. Franco https://orcid.org/0000-0001-7461-3414

M. Madrid https://orcid.org/0000-0002-7970-2587

J. Vicente-Soler https://orcid.org/0000-0001-8759-6545

J. Cansado https://orcid.org/0000-0002-2342-8152

T. Soto https://orcid.org/0000-0003-2965-318X

### Data availability

The ITS and D1/D2 LSU sequences of representative yeast species generated in this study have been deposited in GenBank under accession numbers PX593007–PX593094 and PX579025–PX579108, respectively. The occurrence data and associated metadata for the culturable yeast isolates have been deposited in GBIF through Pensoft's IPT Data Hosting Centre and are available at https://doi.org/10.15468/c3yb63. Additional data supporting the enzymatic activity and stress-tolerance analyses are provided in the Supplementary Information.

## Supplementary materials

Supplementary material 1 Molecular identification and distribution of yeast species isolated Data type docx https://binary.pensoft.net/file/1662063This dataset is made available under the Open Database License (http://opendatacommons.org/licenses/odbl/1.0/). The Open Database License (ODbL) is a license agreement intended to allow users to freely share, modify, and use this Dataset while maintaining this same freedom for others, provided that the original source and author(s) are credited. Alejandra Martínez-Rodrigo, Andrés Núñez, Alejandro Franco, Marisa Madrid, Jero Vicente-Soler, José Cansado, Teresa Soto

Supplementary material 2 Richness and diversity indices by zone and season for seawater and sediment samples Data type docx https://binary.pensoft.net/file/1662064This dataset is made available under the Open Database License (http://opendatacommons.org/licenses/odbl/1.0/). The Open Database License (ODbL) is a license agreement intended to allow users to freely share, modify, and use this Dataset while maintaining this same freedom for others, provided that the original source and author(s) are credited. Alejandra Martínez-Rodrigo, Andrés Núñez, Alejandro Franco, Marisa Madrid, Jero Vicente-Soler, José Cansado, Teresa Soto

Supplementary material 3 SIMPER analysis of contributions of culturable yeast species between seasons Data type docx https://binary.pensoft.net/file/1662065This dataset is made available under the Open Database License (http://opendatacommons.org/licenses/odbl/1.0/). The Open Database License (ODbL) is a license agreement intended to allow users to freely share, modify, and use this Dataset while maintaining this same freedom for others, provided that the original source and author(s) are credited. Alejandra Martínez-Rodrigo, Andrés Núñez, Alejandro Franco, Marisa Madrid, Jero Vicente-Soler, José Cansado, Teresa Soto

Supplementary material 4 Redundancy analysis (RDA) for the relationship between physicochemical parameters and yeast community structure Data type pdf https://binary.pensoft.net/file/1662066This dataset is made available under the Open Database License (http://opendatacommons.org/licenses/odbl/1.0/). The Open Database License (ODbL) is a license agreement intended to allow users to freely share, modify, and use this Dataset while maintaining this same freedom for others, provided that the original source and author(s) are credited. Alejandra Martínez-Rodrigo, Andrés Núñez, Alejandro Franco, Marisa Madrid, Jero Vicente-Soler, José Cansado, Teresa Soto

Supplementary material 5 Stress-response profiles under multiple stressors of representative strains Data type pdf https://binary.pensoft.net/file/1662067This dataset is made available under the Open Database License (http://opendatacommons.org/licenses/odbl/1.0/). The Open Database License (ODbL) is a license agreement intended to allow users to freely share, modify, and use this Dataset while maintaining this same freedom for others, provided that the original source and author(s) are credited. Alejandra Martínez-Rodrigo, Andrés Núñez, Alejandro Franco, Marisa Madrid, Jero Vicente-Soler, José Cansado, Teresa Soto

Supplementary material 6 Profiles of extracellular enzymatic activities in representative strains of the isolated yeast species Data type pdf https://binary.pensoft.net/file/1662068This dataset is made available under the Open Database License (http://opendatacommons.org/licenses/odbl/1.0/). The Open Database License (ODbL) is a license agreement intended to allow users to freely share, modify, and use this Dataset while maintaining this same freedom for others, provided that the original source and author(s) are credited. Alejandra Martínez-Rodrigo, Andrés Núñez, Alejandro Franco, Marisa Madrid, Jero Vicente-Soler, José Cansado, Teresa Soto
