# Supplementary material for: Unveiling the diversity, ecology, and biotechnological potential of culturable marine yeasts in Western Mediterranean coastal ecosystems
Source: IMA Fungus. 2026 May 29;17:e182209. doi: 10.3897/imafungus.17.182209 (PMC13241915; doi:10.3897/imafungus.17.182209)
Supplement: Supplementary material 1 — Molecular identification and distribution of yeast species isolated [file imafungus-17-e182209-s001.docx]

**Table S1.** Molecular identification and distribution of culturable yeast species isolated from different zones of Southeastern Mediterranean coast of Spain.

| **Identify species** | **Representative strain Nº** | **GenBank access Nº** | | **Isolation site (source)** | **BLAST Identity (%) – ITS (NCBI Accession) *** | **BLAST Identity (%) - D1/D2 (NCBI Accession) *** | ***Phylum*** |
| --- | --- | --- | --- | --- | --- | --- | --- |
|  |  | **ITS region** | **D1/D2 region** |  |  |  |  |
| *Aureobasidium melanogenum* | PA36-76 | PX593008 | PX579025 | Cabo de Palos (seawater) | 99.81%  (NR_159598.1) | 100%  (NG_056960.1) | *Ascomycota* |
| *Aureobasidium pullulans* | HA11-57 | PX593009 | PX579026 | La Azohía (seawater) | 99.31%  (NR_144909.1) | 100% (NG_055734.1) | *Ascomycota* |
| *Aureobasidium* sp. | GA23-246 | PX593007 | Not available | Águilas Port (seawater) | 97.87% (NR_189918.1) | Not available | *Ascomycota* |
| *Australozyma* sp*.* | MA16-224 | PX593010 | PX579027 | Mar Menor (seawater) | 95.96%  (KX781276.1) | 94.18% (NG_058986.1) | *Ascomycota* |
| *Candida mengyuniae* | CA26-165 | PX593013 | PX579030 | Cartagena Port (seawater) | 99.64%  (NR_159549.1) | 100%  (NG_064316.1) | *Ascomycota* |
| *Candida oleophila* | PA68-428 | PX593014 | Not available | Cabo de Palos (seawater) | 99.08%  (NR_155224.1) | Not available | *Ascomycota* |
| *Candida palmioleophila* | CA11-29 | PX593015 | PX579031 | Cartagena Port (seawater) | 98.53%  (NR_077076.1) | 100%  (NG_054846.1) | *Ascomycota* |
| *Candida parapsilosis* | GS29-371 | PX593016 | PX579032 | Águilas Port (marine sediment) | 100%  (NR_130673.1) | 99.82%  (NG_054833.1) | *Ascomycota* |
| *Candida psychrophila* | GA9-4 | PX593017 | PX579033 | Águilas Port (seawater) | 99.43%  (NR_151805.1) | 100%  (NG_060823.1) | *Ascomycota* |
| *Candida railenensis* | HS3-273 | PX593018 | Not available | La Azohía (marine sediment) | 99.63%  (NR_077080.1) | Not available | *Ascomycota* |
| *Candida zeylanoides* | CS19-178 | PX593019 | PX579034 | Cartagena Port (marine sediment) | 99.8%  (NR_131278.1) | 100% (NG_060834.1) | *Ascomycota* |
| *Candidozyma haemuli* | CA100-387 | PX593020 | PX579035 | Cartagena Port (seawater) | 98.91%  (NR_130669.1) | 99.80%  (NG_060809.1) | *Ascomycota* |
| *Clavispora* sp. | MA8-99 | PX593021 | PX579036 | Mar Menor (seawater) | 97.02%  (NR_130677.1) | 95.48%  (NG_055408.1) | *Ascomycota* |
| *Cyberlindnera rhodanensis* | GA16-131 | PX593022 | PX579037 | Águilas Port (seawater) | 99.82%  (PX349424.1) | 99.64%  (NG_058753.1) | *Ascomycota* |
| *Cystobasidium slooffiae* | PA3-72 | PX593023 | PX579038 | Cabo de Palos (seawater) | 97.69%  (NR_103568.1) | 99.66%  (NG_059008.1) | *Ascomycota* |
| *Debaryomyces fabryi* | CA31-166 | PX593024 | PX579039 | Cartagena Port (seawater) | 100%  (NR_138186.1) | Not available | *Ascomycota* |
| *Debaryomyces hansenii* | OA4-85 | PX593025 | PX579040 | La Manga (seawater) | 99.12%  (NR_120016.1) | 100%  (NG_042634.1) | *Ascomycota* |
| *Debaryomyces prosopidis* | LA5-110 | PX593026 | PX579041 | Salt ponds (seawater) | 99.82%  (NR_077067.1) | 99.84%  (NG_055701.1) | *Ascomycota* |
| *Debaryomyces subglobosus* | LA3-108 | PX593027 | PX579042 | Salt ponds (seawater) | 100%  (KY103295.1) | 99.84%  (NG_055699.1) | *Ascomycota* |
| *Debaryomyces vindobonensis* | LS8-122 | PX593028 | PX579043 | Salt ponds (marine sediment) | 100%  (NR_138218.1) | 100%  (NG_055692.1) | *Ascomycota* |
| *Diutina catenulata* | GS7-11 | PX593029 | PX579044 | Águilas Port (marine sediment) | 100%  (NR_077200.1) | 99.78%  (NG_059158.1) | *Ascomycota* |
| *Exophiala oligosperma* | UA16-127 | PX593030 | PX579045 | Portmán (seawater) | 99.82%  (NR_111134.1) | 99.64%  (NG_059201.1) | *Ascomycota* |
| *Filobasidium*  *chernovii* | UA66-456 | PX593031 | PX579046 | Portmán (seawater) | 98.78%  (NR_073223.1) | 99.62%  (NG_068965.1) | *Basidiomycota* |
| *Filobasidium*  *magnum* | GA12-6 | PX593032 | PX579047 | Águilas Port (seawater) | 100%  (NR_130655.1) | 100%  (NG_069409.1) | *Basidiomycota* |
| *Filobasidium*  *oeirense* | PS18-336 | PX593033 | PX579048 | Cabo de Palos (marine sediment) | 99.12%  (NR_077106-1) | 99.11%  (NG_070508.1) | *Basidiomycota* |
| *Filobasidium uniguttulatum* | CS3-33 | PX593034 | PX579049 | Cartagena Port (marine sediment) | 97.91%  (NR_111070.1) | 99.83%  (NG_056269.1) | *Basidiomycota* |
| *Fonsecazyma* sp. | CAYG01-125 | PX593035 | Not available | Cartagena Port (seawater) | 86.76%  (NR_137814.1) | Not available | *Basidiomycota* |
| *Geotrichum pandrosioniae* | ZA49-269 | PX593036 | PX579050 | Calnegre (seawater) | 100%  (NR_189975.1) | 99.24%  (NG_242136.1) | *Ascomycota* |
| *Geotrichum* sp. | CA92-292 | PX593037 | PX579051 | Cartagena Port (seawater) | 97.44%  (NR_198341.1) | 98.64%  (NG_243798.1) | *Ascomycota* |
| *Hanseniaspora guilliermondii* | MA5-96 | PX593038 | PX579052 | Mar Menor (seawater) | 99.85%  (NR_138192.1) | 100%  (NG_055420.1) | *Ascomycota* |
| *Hanseniaspora* sp. | HA27-277 | PX593039 | PX579053 | La Azohía (seawater) | 97.67%  (NR_155181.1) | 99.28%  (NG_058305.1) | *Ascomycota* |
| *Hortaea werneckii* | CS6-36 | PX593040 | PX579054 | Cartagena Port (marine sediment) | 99.07%  (NR_145338.1) | 99.27%  (NG_057773.1) | *Ascomycota* |
| *Hyphopichia burtonii* | MA19-237 | PX593041 | PX579055 | Mar Menor (seawater) | 99.17%  (NR_111253.1) | 99.60%  (NG_054819.1) | *Ascomycota* |
| *Kuraishia molischiana* | MS20-452 | PX593042 | PX579056 | Mar Menor (marine sediment) | 98.85%  (NR_164409.1) | 99.81%  (NG_058325.1) | *Ascomycota* |
| *Kwoniella dendrophila* | ZA5-13 | PX593043 | PX579057 | Calnegre (seawater) | 99.58%  (NR_073257.1) | 98.11%  (NG_058326.1) | *Basidiomycota* |
| *Lachancea thermotolerans* | MA4-95 | PX593044 | PX579058 | Mar Menor (seawater) | 98.93%  (NR_111334.1) | 100%  (NG_42626.1) | *Ascomycota* |
| *Metschnikowia* sp. | GS22-255 | PX593045 | PX579059 | Cartagena Port (marine sediment) | 93.06%  (NR_158858.1) | 91.52%  (NG_064475.1) | *Ascomycota* |
| *Metschnikowia viticola* | BA24-67 | PX593046 | PX579060 | Calbanque (seawater) | 99.71%  (NR_077083.1) | 99.40%  (NG_058346.1) | *Ascomycota* |
| *Meyerozyma caribbica* | LS1-115 | PX593047 | PX579061 | Salt ponds (marine sediment) | 99.81%  (NR_149348.1) | 99.39%  (NG_054806.1) | *Ascomycota* |
| *Meyerozyma carpophila* | GA1-1 | PX593048 | PX579062 | Águilas Port (seawater) | 99.79%  (NR_152984.1) | 99.63%  (NG_069408.1) | *Ascomycota* |
| *Meyerozyma guilliermondii* | PS7-207 | PX593049 | PX579063 | Cabo de Palos (marine sediment) | 99.81%  (NR_111247.1) | 99.82%  (NG_042640.1) | *Ascomycota* |
| *Naganishia albida* | GA2-2 | PX593050 | PX579064 | Águilas Port (seawater) | 99.61%  (NR_111046.1) | 100%  (NG_055717.1) | *Basidiomycota* |
| *Naganishia bhutanensis* | LS13-365 | PX593051 | PX579065 | Salt ponds (marine sediment) | 99.40%  (NR_077082.1) | 99.64%  (NG_058350.1) | *Basidiomycota* |
| *Naganishia diffluens* | PA31-75 | PX593052 | PX579066 | Cabo de Palos (seawater) | 99.45%  (NR_111051.1) | 99.42%  (NG_058351.1) | *Basidiomycota* |
| *Naganishia liquefaciens* | BS2-234 | PX593053 | PX579067 | Calbanque (seawater) | 98.56%  (NR_073220.1) | 99.83%  (NG_057655.1) | *Basidiomycota* |
| *Naganishia nivalis* | PA39-202 | PX593054 | PX579068 | Cabo de Palos (seawater) | 98.73%  (NR_197467.1) | 99.8%  (NG_079496.1) | *Basidiomycota* |
| *Naganishia uzbekistanensis* | MS4-103 | PX593055 | PX579069 | Mar Menor (marine sediment) | 97.82%  (NR_073219.1) | 99.66%  (NG_067254.1) | *Basidiomycota* |
| *Nakaseomyces glabratus* | PA58-423 | PX593056 | PX579070 | Cabo de Palos (seawater) | 96.27%  (NR_130691.1) | 99.82%  (NG_055062.1) | *Basidiomycota* |
| *Ogataea zsoltii* | UA3-39 | PX593057 | PX579071 | Portmán (seawater) | 99.39%  (NR_138176.1) | 99.63%  (NG_055088.1) | *Ascomycota* |
| *Papiliotrema fonsecae* | OA17-211 | PX593058 | Not available | La Manga (seawater) | 100%  (NR_119972.1) | Not available | *Basidiomycota* |
| *Papiliotrema mangalensis* | BA73-320 | PX593059 | PX579073 | Calbanque (seawater) | 99.06%  (NR_144816.1) | 99.31%  (NG_057803.1) | *Basidiomycota* |
| *Papiliotrema terrestris* | GA28-251 | PX593060 | PX579074 | Águilas Port (seawater) | 99.51%  (NR_073350.1) | 99.15%  (NG_058367.1) | *Basidiomycota* |
| *Pichia kudriavzevii* | CA72-286 | PX593061 | PX579075 | Cartagena Port (seawater) | 100%  (NR_131315.1) | 99.63%  (NG_055104.1) | *Ascomycota* |
| *Pichia norvegensis* | BS7-326 | Not available | PX579076 | Cartagena Port (marine sediment) | Not available | 99.64%  (NG_055113.1) | Ascomycota |
| *Pichia pseudolambica* | MA12-354 | PX593062 | PX579077 | Mar Menor (seawater) | 93.31%  (NR_153281.1) | 99.63%  (NG_060822.1) | *Ascomycota* |
| *Pichia* sp1. | GA15-130 | PX593063 | PX579078 | Águilas Port (seawater) | 96.86%  (NR_17335) | 91.06%  (NG_075177.1) | *Ascomycota* |
| *Pichia* sp2. | GA31-367 | PX593064 | PX579079 | Águilas Port (seawater) | 99.08%  (NR_138243.1) | 95.32%  (NG_055115.1) | *Ascomycota* |
| *Pseudozyma hubeiensis* | HA18-153 | PX593065 | PX579080 | La Azohía (seawater) | 98.58%  (NR_137546.1) | 97.75%  (NG_067794.1) | *Basidiomycota* |
| *Pseudozyma pruni* | CA96-383 | PX593066 | PX579081 | Cartagena Port (seawater) | 99.27%  (NR_165983.1) | 97.41%  (NG_058380.1) | *Basidiomycota* |
| *Rhodosporidiobolus fluvialis* | ZS4-148 | PX593067 | PX579082 | Calnegre (marine sediment) | 98.52%  (NR_077089.1) | 99.40%  (NG_042341.1) | *Basidiomycota* |
| *Rhodotorula alborubescens* | OA1-82 | PX593068 | PX579083 | La Manga (seawater) | 99.58%  (NR_153197.1) | 100%  (NG_068967.1) | *Basidiomycota* |
| *Rhodotorula diobovata* | CA9-27 | PX593069 | PX579084 | Cartagena Port (seawater) | 99.64%  (NR_073271.1) | 99.82%  (NG_042340.1) | *Basidiomycota* |
| *Rhodotorula mucilaginosa* | ZA6-52 | PX593070 | PX579085 | Calnegre (seawater) | 99.10%  (NR_073296.1) | 99.82%  (NG_055716.1) | *Basidiomycota* |
| *Sampaiozyma ingeniosa* | MA23-349 | PX593071 | PX579086 | Mar Menor (seawater) | 94.68%  (NR_111080.1) | 99.46% (NG_058398.1) | *Basidiomycota* |
| *Sampaiozyma vanillica* | CA53-177 | PX593072 | PX579087 | Cartagena Port (seawater) | 98.12%  (NR_073315.1) | 99.82% (NG_058399.1) | *Basidiomycota* |
| *Saturnispora* sp. | HA15-150 | Not available | PX579088 | La Azohía (seawater) | Not available | 86.89% (NG_055182.1) | *Ascomycota* |
| *Scheffersomyces spartinae* | MA18-226 | PX593073 | PX579089 | Mar Menor (seawater) | 100%  (NR_111290.1) | 99.63%  (NG_042639.1) | *Ascomycota* |
| *Sporobolomyces reniformis* | UA41-304 | PX593074 | PX579090 | Portmán (seawater) | 99.26%  (NR_174786.1) | Not available | *Basidiomycota* |
| *Starmerella lactis-condensi* | UA44-306 | PX593075 | PX579091 | Portmán (seawater) | 97.10%  (NR_155822.1) | 99.85%  (NG_060813.1) | *Ascomycota* |
| *Symmetrospora marina* | UA13-46 | PX593076 | PX579092 | Portmán (seawater) | 98.47%  (NR_073272.1) | 100%  (MT756564.1) | *Basidiomycota* |
| *Symmetrospora* sp. | BA81-461 | PX593077 | Not available | Calbanque (seawater) | 93.73%  (NR_171799.1) | Not available | *Basidiomycota* |
| *Torulaspora delbrueckii* | MS10-227 | PX593078 | PX579093 | Mar Menor (marine sediment) | 98.02%  (NR_111257.1) | 99.46% (NG_058413.1) | *Ascomycota* |
| *Trichosporon aquatile* | PA47-329 | PX593079 | PX579094 | Cabo de Palos (seawater) | 98.95%  (NR_155872.1) | 99.64%  (NG_058417.1) | *Basidiomycota* |
| *Vishniacozyma carnescens* | LA20-465 | PX593080 | PX579095 | Salt ponds (seawater) | 98.69%  (NR_130695.1) | 99.27% (NG_058430.1) | *Basidiomycota* |
| *Vishniacozyma foliicola* | HA36-377 | PX593081 | PX579096 | La Azohía (seawater) | 97.78%  (NR_144809.1) | 99.46%  (NG_067769.1) | *Basidiomycota* |
| *Vishniacozyma pseudocarnescens* | BA54-198 | PX593083 | PX579097 | Calbanque (seawater) | 99.11%  (NR_198531.1) | 98.28%  (NG_243327.1) | *Basidiomycota* |
| *Vishniacozyma* sp. | CA84-290 | PX593082 | Not available | Cartagena Port (seawater) | 97.35%  (NR_175761.1) | Not available | *Basidiomycota* |
| *Vishniacozyma tephrensis* | HA32-374 | PX593084 | PX579098 | La Azohía (seawater) | 99.11%  (NR_144812.1) | Not available | *Basidiomycota* |
| *Vishniacozyma victoriae* | LS9-232 | PX593085 | PX579099 | Salt ponds (marine sediment) | 99.30%  (NR_073260.1) | 99.48%  (NG_057678.1) | *Basidiomycota* |
| *Wickerhamiella osmotolerans* | CA34-167 | PX593086 | PX579100 | Cartagena Port (seawater) | 98.40%  (NR_172734.1) | 99.43% (NG_075243.1) | *Ascomycota* |
| *Wickerhamiella tropicalis* | CA63-283 | PX593087 | PX579101 | Cartagena Port (seawater) | 98.72%  (NR_172735.1) | 98.47% (NG_075244.1) | *Ascomycota* |
| *Wickerhamomyces anomalus* | OA15-210 | PX593088 | PX579102 | La Manga (seawater) | 98.80%  (NR_111210.1) | 99.82%  (NG_057174.1) | *Ascomycota* |
| *Yamadazyma atlantica* | CS57-394 | PX593011 | PX579028 | Cartagena Port (marine sediment) | 99.38%  (NR_155983.1) | 99.6%  (NG_054857.1) | *Ascomycota* |
| *Yamadazyma atmosphaerica* | PS12-235 | PX593012 | PX579029 | Cabo de Palos (marine sediment) | 99.64%  (AJ539369.1) | 99.80% (NG_054853.1) | *Ascomycota* |
| *Yamadazyma epiphylla* | UA12-45 | PX593089 | PX579103 | Portmán (seawater) | 100%  (NR_185526.1) | Not available | *Ascomycota* |
| *Yamadazyma mexicana* | UA6-42 | PX593090 | PX579104 | Portmán (seawater) | 99.80%  (NR_138213.1) | 99.81%  (NG_058439.1) | *Ascomycota* |
| *Yarrowia deformans* | BA65-313 | PX593091 | PX579105 | Portmán (seawater) | 99.66%  (NR_161005.1) | 100%  (NG_067515.1) | *Ascomycota* |
| *Yarrowia lipolytica* | CA42-174 | PX593092 | PX579106 | Cartagena Port (seawater) | 99.63%  (NR_111212.1) | 99.79% (NG_055393.1) | *Ascomycota* |
| *Zalaria alba* | BA34-69 | PX593093 | PX579107 | Calbanque (seawater) | 99.35%  (NR_153465.1) | 98.42% (NG_060009.1) | *Ascomycota* |
| *Zygoascus polysorbophilus* | CA3-24 | PX593094 | PX579108 | Cartagena Port (seawater) | 99.57%  (NR_160311.1) | 98.9% (NG_064312.1) | *Ascomycota* |

(*) GenBank NCBI accession number of the type strains of the different species isolated
