# Supplementary material for: Unveiling the diversity, ecology, and biotechnological potential of culturable marine yeasts in Western Mediterranean coastal ecosystems
Source: IMA Fungus. 2026 May 29;17:e182209. doi: 10.3897/imafungus.17.182209 (PMC13241915; doi:10.3897/imafungus.17.182209)
Supplement: Supplementary material 2 — Richness and diversity indices by zone and season for seawater and sediment samples [file imafungus-17-e182209-s002.docx]

|  | **Seawater** | | | | | | **Sediment** | | | | | |
| --- | --- | --- | --- | --- | --- | --- | --- | --- | --- | --- | --- | --- |
| **Zones** | **Individuals** | **Observed spp.** | **Margalef** | **Simpson** | **Shannon** | **Chao1** | **Individuals** | **Observed sp.** | **Margalef** | **Simpson** | **Shannon** | **Chao1** |
| Port of Águilas | 23 | 13 | 3.83 | 0.88 | 2.34 | 20 | 17 | 8 | 2.47 | 0.83 | 1.92 | 8.75 |
| Calnegre | 19 | 9 | 2.72 | 0.84 | 2.01 | 10.5 | 8 | 5 | 1.92 | 0.78 | 1.56 | 5.25 |
| La Azohía | 23 | 14 | 4.15 | 0.9 | 2.45 | 36.5 | 8 | 7 | 2.89 | 0.84 | 1.91 | 14.25 |
| Port of Cartagena | 50 | 27 | 6.39 | 0.93 | 2.96 | 64.2 | 29 | 15 | 4.45 | 0.89 | 2.51 | 34.3 |
| Portmán | 34 | 16 | 4.25 | 0.88 | 2.48 | 28 | 9 | 4 | 1.36 | 0.67 | 1.21 | 5 |
| Calblanque | 30 | 14 | 3.82 | 0.83 | 2.21 | 26 | 10 | 4 | 1.74 | 0.68 | 1.36 | 6.5 |
| Cabo de Palos | 28 | 14 | 3.9 | 0.86 | 2.41 | 18.2 | 15 | 9 | 2.95 | 0.86 | 2.08 | 12.3 |
| La Manga | 34 | 13 | 3.4 | 0.9 | 2.42 | 13.5 | 4 | 4 | 2.16 | 0.75 | 1.39 | 10 |
| Mar Menor | 25 | 13 | 3.73 | 0.86 | 2.25 | 58 | 18 | 7 | 2.07 | 0.76 | 1.64 | 13 |
| Salt ponds | 19 | 8 | 2.38 | 0.84 | 1.94 | 9.5 | 12 | 9 | 3.62 | 0.87 | 2.21 | 46 |
| **Seasons** | **Individuals** | **Observed spp.** | **Margalef** | **Simpson** | **Shannon** | **Chao1** | **Individuals** | **Observed spp.** | **Margalef** | **Simpson** | **Shannon** | **Chao1** |
| Autumn | 78 | 32 | 7.12 | 0.9 | 2.89 | 109 | 29 | 16 | 3.9 | 0.88 | 2.38 | 2.76 |
| Winter | 69 | 27 | 6.14 | 0.93 | 2.93 | 40 | 30 | 19 | 5.29 | 0.92 | 2.76 | 64.5 |
| Spring | 68 | 32 | 7.35 | 0.94 | 3.13 | 56.4 | 41 | 14 | 4.01 | 0.9 | 2.5 | 25.3 |
| Summer | 70 | 24 | 5.41 | 0.9 | 2.7 | 37.2 | 30 | 11 | 2.94 | 0.83 | 2.06 | 14.33 |

**Table S2.** Richness and diversity indices by zone and season for seawater and sediment samples
