# Supplementary material for: Unveiling the diversity, ecology, and biotechnological potential of culturable marine yeasts in Western Mediterranean coastal ecosystems
Source: IMA Fungus. 2026 May 29;17:e182209. doi: 10.3897/imafungus.17.182209 (PMC13241915; doi:10.3897/imafungus.17.182209)
Supplement: Supplementary material 3 — SIMPER analysis of contributions of culturable yeast species between seasons [file imafungus-17-e182209-s003.docx]

**Table S3.** SIMPER analysis of yeast species contributing to dissimilarities between seasons

| **Species** | **average** | **overall** | **sd** | **ratio** | **ava** | **avb** | **ord** | **cusum** | ***p*** |
| --- | --- | --- | --- | --- | --- | --- | --- | --- | --- |
| **Autumn-Winter** |  | | | | | | | | |
| *Naganishia diffluens* | 0.034 | 0.804 | 0.043 | 0.790 | 0.0 | 0.4 | 21 | 0.896 | 0.018 |
| **Autumn-Spring** |  | | | | | | | | |
| *Metschnikowia* sp. | 0.043 | 0.885 | 0.047 | 0.918 | 0.0 | 0.5 | 87 | 0.804 | 0.002 |
| *Papiliotrema terrestris* | 0.027 | 0.885 | 0.040 | 0.670 | 0.1 | 0.3 | 49 | 0.936 | 0.022 |
| *Rhodotorula mucilaginosa* | 0.061 | 0.885 | 0.055 | 1.109 | 1.0 | 0.4 | 6 | 1.000 | 0.012 |
| **Autumn-Summer** |  | | | | | | | | |
| *Aureobasidium melanogenum* | 0.041 | 0.822 | 0.050 | 0.810 | 0.3 | 0.3 | 27 | 0.120 | 0.017 |
| *Candida parapsilosis* | 0.046 | 0.822 | 0.048 | 0.954 | 0.2 | 0.5 | 24 | 0.426 | 0.036 |
| *Diutina catenulata* | 0.021 | 0.822 | 0.037 | 0.576 | 0.2 | 0.1 | 64 | 0.690 | 0.035 |
| *Fonsecazyma* sp*.* | 0.015 | 0.822 | 0.030 | 0.490 | 0.2 | 0.0 | 77 | 0.766 | 0.029 |
| *Naganishia uzbekistanensis* | 0.016 | 0.822 | 0.033 | 0.482 | 0.2 | 0.0 | 81 | 0.967 | 0.034 |
| **Winter-Spring** |  | | | | | | | | |
| *Aureobasidium* sp. | 0.041 | 0.876 | 0.049 | 0.846 | 0.4 | 0.3 | 66 | 0.059 | 0.022 |
| *Metschnikowia* sp. | 0.043 | 0.876 | 0.047 | 0.913 | 0.0 | 0.5 | 67 | 0.872 | 0.002 |
| *Naganishia diffluens* | 0.034 | 0.876 | 0.043 | 0.775 | 0.4 | 0.0 | 28 | 0.931 | 0.032 |
| **Winter-Summer** |  | | | | | | | | |
| *Aureobasidium* sp. | 0.040 | 0.847 | 0.051 | 0.777 | 0.4 | 0.0 | 66 | 0.058 | 0.028 |
| *Candida parapsilosis* | 0.045 | 0.847 | 0.048 | 0.945 | 0.2 | 0.5 | 53 | 0.466 | 0.047 |
| *Naganishia diffluens* | 0.038 | 0.847 | 0.046 | 0.818 | 0.4 | 0.1 | 81 | 0.955 | 0.002 |
| *Papiliotrema fonsecae* | 0.030 | 0.847 | 0.046 | 0.655 | 0.2 | 0.2 | 89 | 1.000 | 0.033 |
| *Rhodotorula diobovata* | 0.036 | 0.847 | 0.051 | 0.714 | 0.2 | 0.3 | 31 | 1.000 | 0.045 |
| *Scheffersomyces spartinae* | 0.016 | 0.847 | 0.032 | 0.491 | 0.2 | 0.0 | 39 | 1.000 | 0.019 |
| **Spring-Summer** |  | | | | | | | | |
| *Candida parapsilosis* | 0.046 | 0.889 | 0.049 | 0.929 | 0.1 | 0.5 | 55 | 0.419 | 0.037 |
| *Candida railenensis* | 0.019 | 0.889 | 0.040 | 0.471 | 0.2 | 0.0 | 45 | 0.465 | 0.014 |
| *Metschnikowia* sp. | 0.046 | 0.889 | 0.051 | 0.907 | 0.5 | 0.0 | 24 | 0.855 | 0.001 |
| *Pichia kudriavzevii* | 0.019 | 0.889 | 0.034 | 0.568 | 0.2 | 0.1 | 91 | 1.000 | 0.028 |
| *Vishniacozyma pseudocarnescens* | 0.039 | 0.889 | 0.047 | 0.821 | 0.4 | 0.2 | 61 | 1.000 | 0.016 |
