# Supplementary material for: Unveiling the diversity, ecology, and biotechnological potential of culturable marine yeasts in Western Mediterranean coastal ecosystems
Source: IMA Fungus. 2026 May 29;17:e182209. doi: 10.3897/imafungus.17.182209 (PMC13241915; doi:10.3897/imafungus.17.182209)
Supplement: Supplementary material 4 — Redundancy analysis (RDA) for the relationship between physicochemical parameters and yeast community structure [file imafungus-17-e182209-s004.pdf]

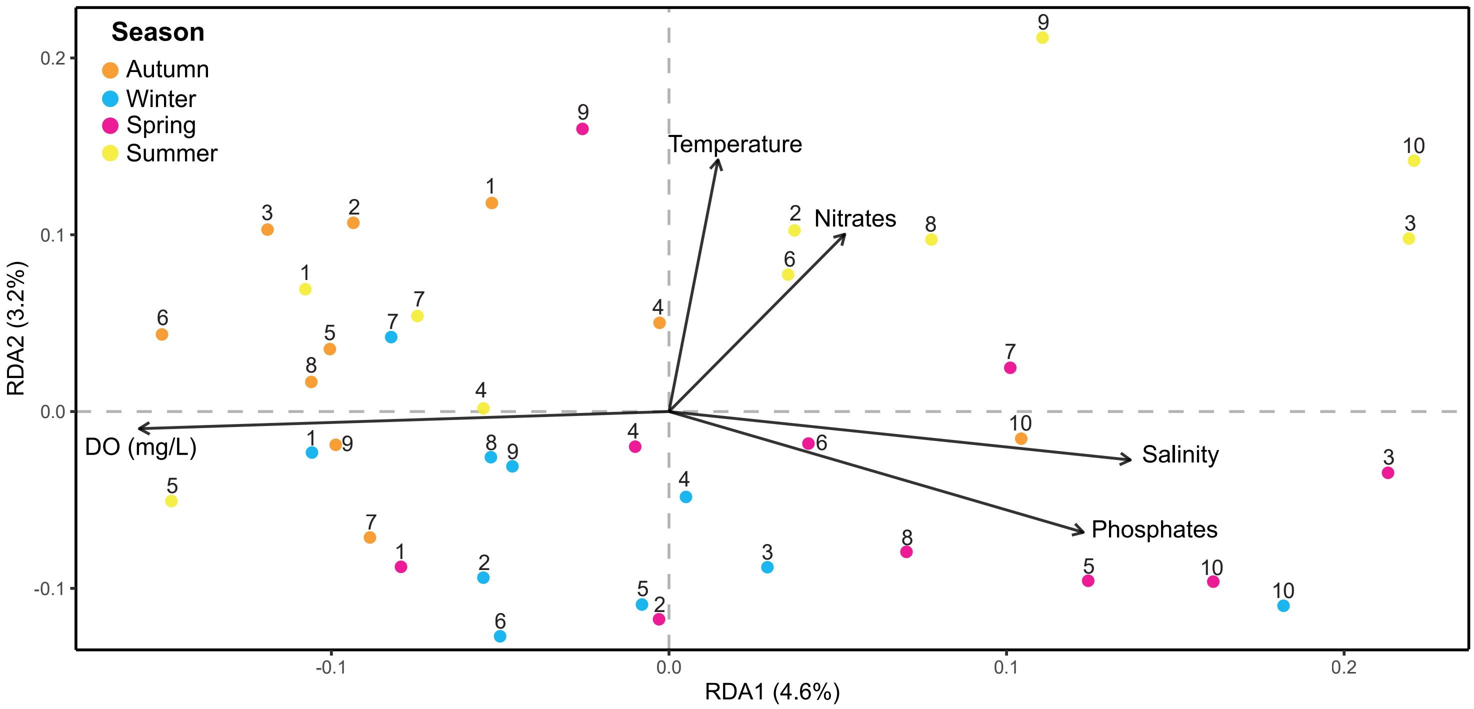

Figure S1. Redundancy analysis (RDA) for the relationship between physicochemical parameters and yeast community structure. The ordination is based on yeast abundances across 10 sampling sites (numbered 1-10) over four seasonal surveys (n=40). Vectors represent the environmental variables; vector length and direction indicate their relative contribution and correlation with the RDA axes. Data points are color-coded by season: orange (Autumn), blue (Winter), pink (Spring), and yellow (Summer). The overall model was statistically significant ($p < 0.05$), with the analysed factors explaining 13% of the total variance (R^2=0.13).
