# Supplementary material for: Unveiling the diversity, ecology, and biotechnological potential of culturable marine yeasts in Western Mediterranean coastal ecosystems
Source: IMA Fungus. 2026 May 29;17:e182209. doi: 10.3897/imafungus.17.182209 (PMC13241915; doi:10.3897/imafungus.17.182209)
Supplement: Supplementary material 5 — Stress-response profiles under multiple stressors of representative strains [file imafungus-17-e182209-s005.pdf]

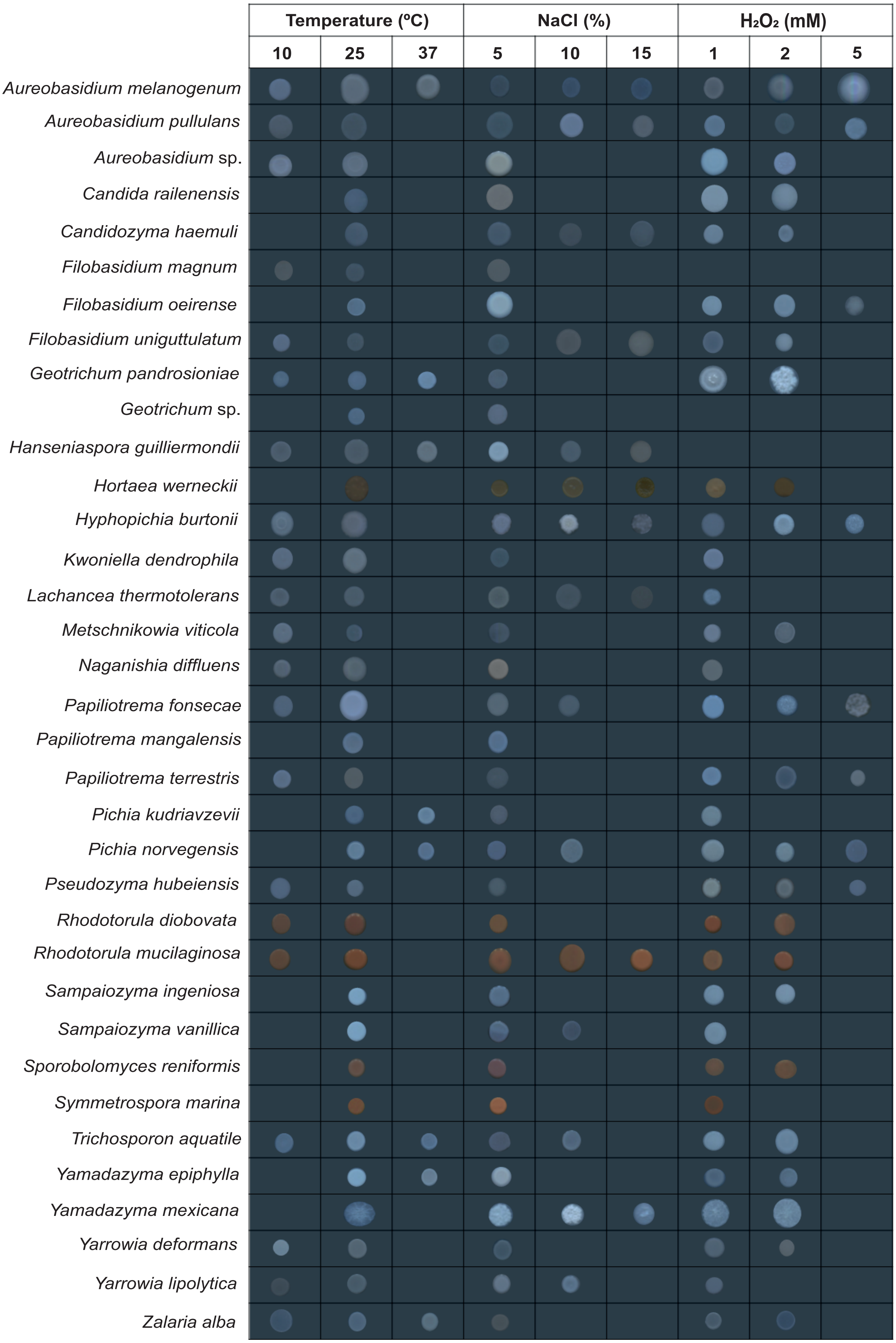

Figure S2. Stress-response profiles of yeast strains under various stress conditions. Growth of representative yeast species in solid media incubated at the indicated temperatures or supplemented with increased concentrations of sodium chloride (salt stress), or hydrogen peroxide (oxidative stress) (complementary to data shown in Table 2).
