# Supplementary material for: Unveiling the diversity, ecology, and biotechnological potential of culturable marine yeasts in Western Mediterranean coastal ecosystems
Source: IMA Fungus. 2026 May 29;17:e182209. doi: 10.3897/imafungus.17.182209 (PMC13241915; doi:10.3897/imafungus.17.182209)
Supplement: Supplementary material 6 — Profiles of extracellular enzymatic activities in representative strains of the isolated yeast species [file imafungus-17-e182209-s006.pdf]

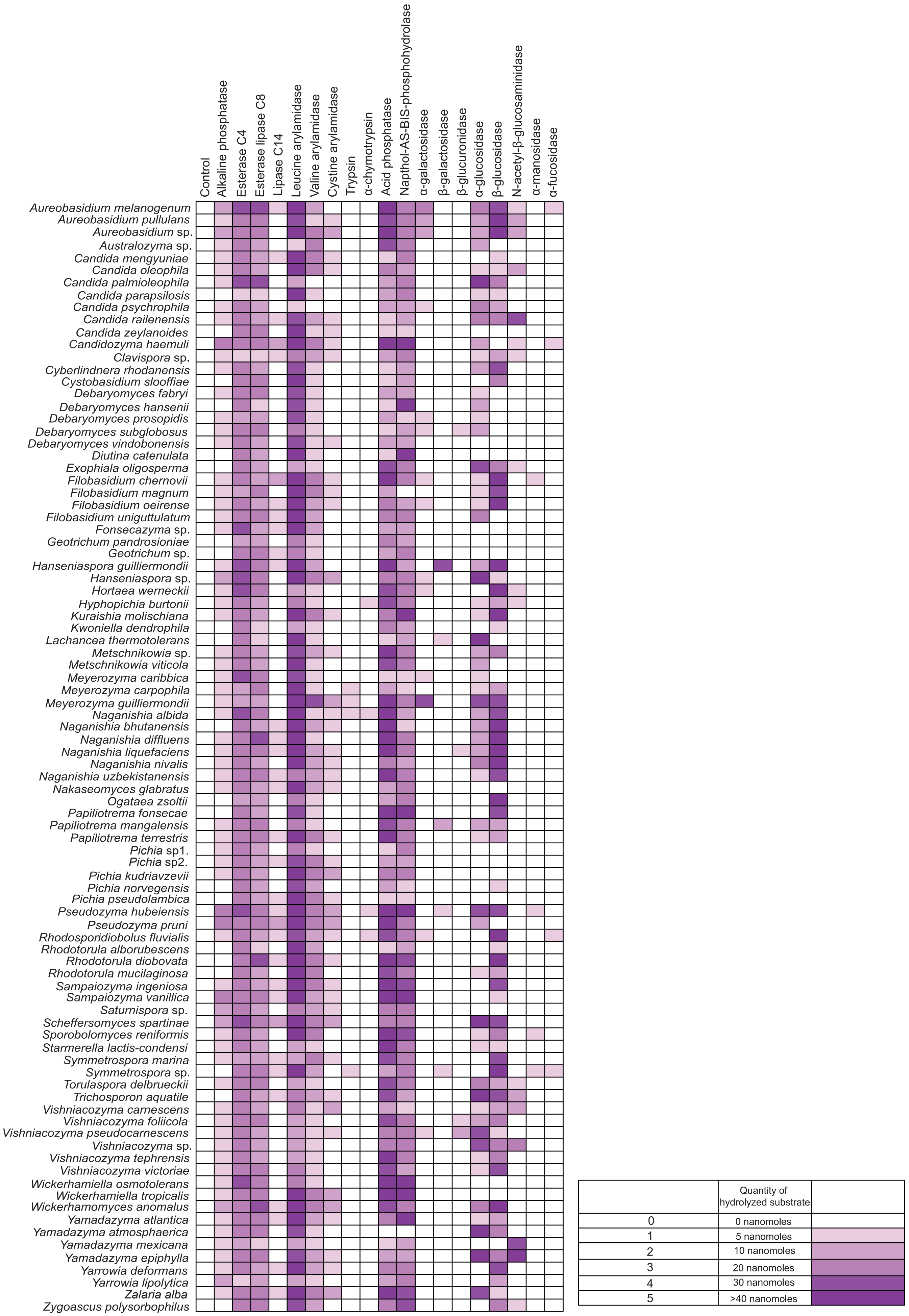

Figure S3. Profiles of extracellular enzymatic activities (API-ZYM, BioMérieux) in representative strains of the isolated yeast species. White squares indicate that no enzymatic activity is detected. An increase in colour intensity represents greater enzymatic activity.
